# CD36 enrichment in HER2-positive mesenchymal stem cells drives therapy refractoriness in breast cancer

**DOI:** 10.1186/s13046-025-03276-z

**Published:** 2025-01-20

**Authors:** Lorenzo Castagnoli, Alma Franceschini, Valeria Cancila, Matteo Dugo, Martina Bigliardi, Claudia Chiodoni, Paolo Toneguzzo, Viola Regondi, Paola A. Corsetto, Filippo Pietrantonio, Serena Mazzucchelli, Fabio Corsi, Antonino Belfiore, Andrea Vingiani, Giancarlo Pruneri, Francesca Ligorio, Mario P. Colombo, Elda Tagliabue, Claudio Tripodo, Claudio Vernieri, Tiziana Triulzi, Serenella M. Pupa

**Affiliations:** 1 Microenvironment and Biomarkers of Solid Tumors Unit, Department of Experimental Oncology, Amadeolab Fondazione IRCCS Istituto Nazionale Dei Tumori Di Milano, Milan, Italy; 2 Tumor Immunology Unit, Department PROMISE, Universita’ Di Palermo, Palermo, Italy; 3 Breast Cancer Unit Clinical Translational and Immunotherapy Research, Department of Medical Oncology, IRCCS Ospedale San Raffaele, Milan, Italy; 4 Molecular Immunology Unit, Department of Experimental Oncology, Amadeolab Fondazione IRCCS Istituto Nazionale Dei Tumori Di Milano, Milan, Italy; 5 Department of Pharmacologicaland, Biomolecular Sciences “Rodolfo Paoletti”, Università Di Milano, Milan, Italy; 6 Medical Oncology Department, Fondazione IRCCS Istituto Nazionale Dei Tumori Di Milano, Milan, Italy; 7 Department of Biomedical and Clinical Sciences, Università Di Milano, Milan, Italy; 8 Surgery Department, Istituti Clinici Scientifici Maugeri IRCCS, Pavia, Italy; 9 Department of Diagnostic Pathology and Laboratory Medicine, Fondazione IRCCS Istituto Nazionale Dei Tumori Di Milano, Milan, Italy; 10 Department of Oncology and Hemato-Oncology, Universita’ di Milano, Milan, Italy; 11 IFOM ETS, The AIRC Institute of Molecular Oncology, Milan, Italy

**Keywords:** HER2, Fatty Acid Uptake, CD36, Cancer Stem Cells, Wnt pathway, Resistance to anti-HER2 therapy

## Abstract

**Background:**

Growing evidence shows that the reprogramming of fatty acid (FA) metabolism plays a key role in HER2-positive (HER2 +) breast cancer (BC) aggressiveness, therapy resistance and cancer stemness. In particular, HER2 + BC has been defined as a "lipogenic disease" due to the functional and bi-directional crosstalk occurring between HER2-mediated oncogenic signaling and FA biosynthesis via FA synthase activity. In this context, the functional role exerted by the reprogramming of CD36-mediated FA uptake in HER2 + BC poor prognosis and therapy resistance remains unclear. In this study, we aimed to elucidate whether enhanced CD36 in mesenchymal HER2 + cancer stem cells (CSCs) is directly involved in anti-HER2 treatment refractoriness in HER2 + BC and to design future metabolism-based approaches targeting both FA reprogramming and the “root” of cancer.

**Methods:**

Molecular, biological and functional characterization of CD36-mediated FA uptake was investigated in HER2 + BC patients, cell lines, epithelial and mesenchymal CSCs. Cell proliferation was analyzed by SRB assay upon treatment with lapatinib, CD36 inhibitor, or Wnt antagonist/agonist. Engineered cell models were generated via lentivirus infection and transient silencing. CSC-like properties and tumorigenesis of HER2 + BC cells with or without CD36 depletion were examined by mammosphere forming efficiency assay, flow cytometry, cell sorting, ALDH activity assay and xenograft mouse model. FA uptake was examined by flow cytometry with FA BODIPY FL C16. Intratumor expression of CSC subsets was evaluated via multiplex immunostaining and immunolocalization analysis.

**Results:**

Molecular data demonstrated that CD36 is significantly upmodulated on treatment in therapy resistant HER2 + BC patients and its expression levels in BC cells is correlated with FA uptake. We provided evidence of a consistent enrichment of CD36 in HER2 + epithelial-mesenchymal transition (EMT)-like CSCs from all tested resistant cell models that mechanistically occurs via Wnt signaling pathway activation. Consistently, both in vitro and in vivo dual blockade of CD36 and HER2 increased the anti-CSC efficacy of anti-HER2 drugs favoring the transition of the therapy resistant mesenchymal CSCs into therapy-sensitive mesenchymal-epithelial transition (MET)-like epithelial state. In addition, expression of CD36 in intratumor HER2 + mesenchymal CSCs is significantly associated with resistance to trastuzumab in HER2 + BC patients.

**Conclusions:**

These results support the metabolo-oncogenic nature of CD36-mediated FA uptake in HER2 + therapy-refractory BC. Our study provides evidence that targeting CD36 might be an effective metabolic therapeutic strategy in the treatment of this malignancy.

**Supplementary Information:**

The online version contains supplementary material available at 10.1186/s13046-025-03276-z.

## Introduction

Breast cancer (BC) is the most commonly diagnosed malignancy in women worldwide [1]. HER2 amplification/overexpression (HER2 +) occurs in approximately 15–20% of BC cases and is a marker of a highly aggressive BC subtype characterized by aggressive clinical behavior [2]. Although pharmacological HER2 inhibition can significantly improve clinical outcomes in all disease stages, primary and acquired resistance to anti-HER2 drugs is a crucial issue, necessitating improved knowledge of HER2 biology [3]. Metabolic reprogramming has recently emerged as a new hallmark of cancer [4]. Recent studies have demonstrated that the reprogramming of cancer cell metabolism is key to supporting the increased proliferative and biosynthetic demands essential for tumor proliferation/aggressiveness [5]. In particular, lipid metabolic remodeling is recognized as a crucial program associated with cancer progression, stemness, and drug resistance [6]. Accordingly, overexpression of lipogenic enzymes has been reported to be a common feature of many cancers [7]. In this peculiar metabolic scenario, fatty acid synthase (FASN), an anabolic enzyme required for de novo biosynthesis of palmitic acid, is overexpressed and activated in 85% of HER2 + BC patients and is significantly associated with poor clinical outcome [7]. Solid preclinical evidence indicates the existence of a positive feedback loop (PFL) of reciprocal stimulation between HER2 and FASN, which couples de novo fatty acid (FA) biosynthesis with oncogenic pathways [5]. Considering the PFL linking HER2 with FASN [8–10], pharmacological inhibition of HER2 could also be sufficient to inhibit FASN activity, thus forcing cancer cells to uptake FAs from the extracellular microenvironment. Consistent with these observations, we hypothesized that the transmembrane FA transporter CD36 could be increased as an adaptive lipid metabolic response to guarantee FAs supply and accumulation within cancer cells when the HER2/FASN oncometabolic PFL is inhibited by anti-HER2 treatments.

Increasing evidence shows that upregulation of CD36 promotes cancer progression, invasiveness, and is negatively correlated with drug responses, patient prognosis and stemness in different oncotypes [11–14].

Reported findings strongly support the identification of a minor neoplastic cell subpopulation, cancer stem cells (CSCs), which have unlimited self-renewal capacity and can switch between different cellular phenotypic states by undergoing epithelial–mesenchymal transition (EMT) or the reverse process, *i.e.*, mesenchymal-epithelial transition (MET) [15]. CSCs contribute significantly to tumor onset, aggressiveness, metastasis, and treatment resistance [16]. Several studies have reported that HER2 is a key regulator of HER2 + CSCs properties [17, 18] and that the clinical efficacy of HER2-specific therapies is related to their ability to target breast CSCs (BCSCs) [17, 19]. Notably, Martin-Castillo et al. showed that aldehyde dehydrogenase-positive (ALDH +) BCSCs that have undergone MET (MET-BCSCs) are responsive to trastuzumab and lapatinib, whereas CD44^High^/CD24^Low^ BCSCs that have undergone EMT (EMT-BCSCs) are resistant to these anti-HER2 agents [20]. Moreover, emerging evidence shows that CSC metabolism relies also on lipid metabolism [21] and, consistent with this relationship, CD36 was found to be enriched in glioblastoma [22] and oral [23] CSCs, suggesting that CSCs rely particularly on dietary lipids to promote tumor aggressiveness, metastasis and therapeutic resistance [14].

In this study, we address the pathobiological role of CD36 in predicting worse clinical outcomes in HER2 + BC patients [13] and in driving therapeutic resistance in HER2 + BC models [11]. Specifically, we provide significant evidence of preferential CD36 upregulation in HER2 + /CD44v6 + EMT-like stem cells via Wnt signaling pathway activation in both preclinical and clinical HER2 + BC models. Elucidating the lipid metabolic pathway(s) underlying CSC maintenance/survival and the mechanism(s) responsible for resistance to targeted therapies could lay the foundation for future innovative approaches for metabolic therapy.

## Materials and methods

### Patient samples

The INT-MI series includes HER2 + BC patients who received neoadjuvant treatment with anthracycline-taxane plus trastuzumab between 2009 and 2018 at the Fondazione IRCCS Istituto Nazionale dei Tumori of Milan, Italy (INT). Samples from these patients were collected at baseline (diagnostic biopsies) and after neoadjuvant chemotherapy (surgical samples). All patients received six cycles of chemotherapy and trastuzumab, administered at 3-week intervals. Matched diagnostic biopsies and surgical samples obtained from 32 patients who did not achieve pathological complete response (pCR) upon treatment were subjected to molecular profiling (Table 1). The samples selected for gene expression profiling (GEP) contained at least 70% tumor cells.

**Table 1:** Clinicopathological characteristics of HER2 + BC patients subjected to molecular profiling analysis

Characteristic	Whole cohort *n* = 32	ΔCD36 + *n* = 20	ΔCD36- *n* = 12
**Median age (IQR)**	47 (40–57)	50 (26–58)	44 (24–54)
**Tumor size**			
T1	1 (3%)	1 (5%)	0
T2	17 (53%)	12 (60%)	5 (42%)
T3	3 (9%)	1 (5%)	2 (16%)
T4	11 (34%)	6 (30%)	5 (42%)
**Lymph node status**			
Positive	24 (75%)	15 (75%)	9 (75%)
Negative	8 (25%)	5 (25%)	3 (25%)
**ER status (10%)**			
Positive	23 (72%)	14 (70%)	9 (75%)
Negative	9 (28%)	6 (30%)	3 (25%)
**PGR status (10%)**			
Positive	17 (53%)	9 (45%)	8 (67%)
Negative	15 (47%)	11 (55%)	4 (33%)
**Ki67 status (20%)**			
Positive	25 (78%)	16 (80%)	9 (75%)
Negative	5 (16%)	4 (20%)	1 (8%)
**Grade**			
II	15 (47%)	10 (50%)	5 (45%)
III	15 (47%)	9 (45%)	6 (50%)
**Chemotherapy**			
**AT-CMF**	25 (78%)	13 (65%)	12 (100%)
**AC-T**	7 (22%)	7 (35%)	0

*Abbreviations*: *A* Anthracycline-taxane, *T* Trastuzumab, *CMF* Cyclophosphamide, methotrexate, fluorouracil

On the other hand, selected nonresponsive (pCR No; *n* = 18) and responsive (pCR Yes; *n* = 18) HER2 + BC patients with similar clinical parameters (Table 2) were subjected to multiplex immunostaining and immunolocalization analyses (see below) to evaluate the intratumor percentage (%) of neoplastic stem-like cells with atypical morphology and the HER2 + /CD44v6 + /CD36 + immunophenotype.

**Table 2:** Clinicopathological characteristics of HER2 + BC patients subjected to analysis of intratumor neoplastic stem-like cell enrichment

Characteristic	pCR Yes *n* = 18	pCR No *n* = 18
**Median age (IQR)**	51 (43, 60)	48 (41, 56)
**Tumor size**		
T1	3 (17%)	1 (6%)
T2	9 (50%)	11 (61%)
T3	3 (17%)	2 (11%)
T4	2 (11%)	4 (22%)
**Lymph node status**		
Positive	12 (67%)	13 (72%)
Negative	6 (33%)	5 (28%)
**ER status (10%)**		
Positive	8 (45%)	16 (88%)
Negative	10 (55%)	2 (12%)
**PGR status (10%)**		
Positive	5 (28%)	9 (50%)
Negative	13 (72%)	9 (50%)
**Ki67 status (20%)**		
Positive	17 (94%)	15 (83%)
Negative	1 (6%)	3 (17%)
**Grade**		
II	6 (33%)	9 (50%)
III	10 (56%)	7 (39%)
**Chemotherapy**		
**AT-CMF**	12 (71%)	12 (67%)
**AC-T**	5 (29%)	6 (33%)

All procedures were performed with the ethical approval of the Independent Ethics Committee of the INT, as stated in the Declarations.

### Gene expression profiling 

Total RNA was extracted from HER2 + BC samples using the miRNeasy Mini Kit (QIAGEN, Hilden, Germany). RNA quality and quantity were assessed using the 2200 TapeStation system (Agilent, Santa Clara, CA, USA) and the Qubit 2.0 Fluorometric Assay (Thermo Fisher Scientific, Waltham, MA, USA), respectively. Gene expression analyses were performed according to the GeneChip WT Pico standard protocols (Affymetrix, Thermo Fisher Scientific). After reverse transcription, cDNA synthesis, amplification, and labeling, the samples were hybridized to human ClariomS arrays for 16 h at 45 °C. After washing and staining using the GeneChip Fluidics Station 450, the arrays were scanned using an Affymetrix Gene Chip Scanner 3000 7G. Primary data were acquired using Affymetrix GeneChip Command Scan Control version 4.0 (developed by Thermo Fisher Scientific). Raw and preprocessed gene expression data are available in the NCBI Gene Expression Omnibus repository under accession number GSE245132.

### Bioinformatic analysis

The raw gene expression data were preprocessed using the gene-level signal space transformation–robust multiarray average (SST-RMA) algorithm implemented in Transcriptome Analysis Console software version 4.0.2 (Thermo Fisher Scientific). Preprocessed data were filtered by removing probe sets without associated Entrez gene identifiers. The genes targeted by multiple probe sets were collapsed by selecting the probe set with the highest variance using the collapseRows R function [24]. Differential expression analysis was performed using the limma package (28,367,255). Preranked gene set enrichment analysis (GSEA) was performed using the fGSEA package [25] on hallmarks pathways (v 7.4), a curated set of lipid metabolism-related Gene Ontology Biological Processes (GOBP, v 7.4) that contains the keywords ‘fatty acid’, ‘lipid’ or ‘triglyceride’ (*n* = 180), cancer stemness-related signaling pathways [26], and CSC signatures [27–30] (Supplementary Table S1). The gene set variation analysis (GSVA) function from the GSVA R package (version 1.50.5) was also applied to the INT samples using the same gene sets to calculate enrichment scores. These scores were then correlated using Pearson correlation. We also analyzed GSE114082 from baseline and Tru-cut biopsies of locally advanced HER2 + BCs from patients in the TRUP window-of-opportunity trial who received one cycle of trastuzumab monotherapy [31]. All bioinformatic analyses were conducted using R software version 4.2.1.

### Tumor cell lines

The human HER2 + BC cell lines were purchased from ATCC (Rockville, MD, USA). BT474 and MDAMB361 cells were grown as monolayer cultures in DMEM (EuroClone, Pero, MI, Italy) supplemented with 10% fetal bovine serum (FBS) (Sigma‒Aldrich, St. Louis, MO, USA), whereas HCC1954, EFM192A, HCC1569, SKBR3, and ZR75.30 cells were grown as monolayer cultures in RPMI1640 medium (EuroClone) supplemented with 10% FBS. The BT474, MDAMB361, HCC1954, SKBR3 and ZR75.30 human tumor cell lines were obtained between 2000 and 2010 and authenticated by short tandem repeat DNA fingerprinting (Eurofins Genomics, Louisville, KY, USA; last verification, January 2022). The HCC1569 and EFM192A cell lines were purchased from ATCC in 2021. All tumor cell lines were cultured in a humidified atmosphere at 37 °C with 5% CO_2_ and were routinely tested and confirmed to remain free of mycoplasma contamination. Mycoplasma contamination was tested using the MycoAlert Plus Kit (Lonza, Basel, Switzerland).

### Flow cytometry and cell sorting

HER2 + BC cell lines were analyzed and sorted based on CD36 expression after incubation with an allophycocyanin (APC)-conjugated anti-CD36 antibody or a matched/corresponding isotype control antibody in the dark at 4 °C for 40 min [clone: 5–271; isotype control: APC Mouse IgG2a κ (Biolegend, San Diego, CA, USA)]. To perform triple immunofluorescence flow cytometry to identify cells with the CD44^High^/CD24^Low^ phenotype, we incubated cells with an APC-conjugated anti-CD36 antibody (BioLegend; clone: 5–271; isotype control: APC Mouse IgG2a κ), a fluorescein-5-isothiocyanate (FITC)-conjugated anti-CD44 antibody (BioLegend) and a PE/Cy7-conjugated antibody [BioLegend; clone: ML5; isotype control: phycoerythrin–cyanine 7 (PE/Cy7) Mouse IgG2a, κ] for 40 min at 4 °C. To perform double immunofluorescence FACS analysis of CD44 and CD24 expression, we incubated cells with an APC-conjugated anti-CD44 antibody (BioLegend) and a PE/Cy7-conjugated antibody (BioLegend; clone: ML5; isotype control: PE/Cy7 Mouse IgG2a, κ) for 40 min at 4 °C. Immunofluorescence analyses were performed using a BD FACSCanto™ II flow cytometer (BD Biosciences, Franklin Lakes, NJ, USA) or after sorting with a high-speed FACSAria II sorter (BD Biosciences) to ensure high purity (> 95%). In all analyses, the data were processed using the FlowJo™ software package (BD Biosciences). The relative median fluorescence intensity (rMFI) and the percentage of positively stained cells were determined by gating on live cells and subtracting the background (isotype) MFI. 7-Aminoactinomycin D (7-AAD) Staining Solution (BD Biosciences) was added before analysis to gate out nonviable cells.

The FA uptake assay was performed as previously described [32]. Cells were seeded at a density of 400,000 cells/well in a 6-well plate for 72 h before the experiments and cultured in serum-free medium supplemented with 2 μM 4,4-Difluoro-5,7-Dimethyl-4-Bora-3a,4a-Diaza-*s*-Indacene-3-Hexadecanoic Acid (BODIPY FL C16; Thermo Fisher Scientific) for 30 min at 37 °C. After washing, the cells were harvested and analyzed via flow cytometry. The MFI was compared between the samples and the internal control condition.

### Cell treatments

Single-cell suspensions of HCC1954, MDAMB361, EFM192A and HCC1569 cells were plated and grown in a 6-well plate at a density of 150,000–400,000 cells/well (according to the cell line) for 72 h at 37 °C with 5% CO_2_. To examine the contribution of FA uptake via CD36 in the formation of HER2 + EMT-like CSCs, HCC1954, MDAMB361, EFM192A and HCC1569 cells were plated and grown in a 6-well plate at a density of 150,000–400,000 cells/well (according to the cell line) and incubated in culture media supplemented with FBS depleted (FBS-FA-) or not (FBS-FA +) of FA for 24 h, 48 h and 72 h. To evaluate the effects of selective WNT inhibition, HCC1954, MDAMB361, EFM192A and HCC1569 cells were treated with the WNT inhibitor LGK974 (Selleckchem, Karl-Schmid-Str.14, Munich, DE) as a single agent at final concentrations of 10 μM and 20 μM (HCC1954, MDAMB361 and EFM192A cells) or 15 μM and 30 μM (HCC1569 cells) or with 0.1% DMSO (Sigma‒Aldrich) as an internal control for 24 h and 48 h at 37 °C with 5% CO_2_. In parallel, to evaluate the effects of selective WNT activation, HCC1954, MDAMB361 and EFM192A cells were treated with the specific WNT agonist SKL2001 (Selleckchem) as a single agent (10, 20 and 40 µM) or with 0.1% DMSO (Sigma‒Aldrich) as an internal control for 24 h. Both the mRNA and protein expression levels of CD36 were measured. To evaluate the effect of CD36 inhibition on CSC subset maintenance, HCC1954, MDAMB361, EFM192A and HCC1569 cells were treated with the CD36 inhibitor sulfo N-succinimidoyl oleate (SSO) (Cayman Chemical, Ann Arbor, MI, USA) as a single agent (100 µM) for 48 h at 37 °C with 5% CO_2_.

### Sulforhodamine B (SRB) cell viability/proliferation assay

To evaluate the antitumor effects of the different drugs, HCC1569, MDAMB361, HCC1954, EFM192A, SKBR3, ZR75.30 and BT474 cells were seeded in 96-well plates at a density of 3,000–5,000 cells per well (according to the cell line) for 72 h before each treatment to allow attachment to the plastic surface. Next, the cells were treated with an increasing concentration gradient of the anti-HER2 lapatinib (Selleckchem) (from 0.05625 μM to 28.8 μM) diluted in DMSO for 24 h, 48 h and 72 h, the WNT antagonist LGK974 (from 5 μM to 80 μM), the WNT agonist SKL2001 (from 5 μM to 80 μM) or the CD36 inhibitor SSO (from 12.5 μM to 400 μM) for different periods (24 h, 48 h and 72 h). Cell viability was assessed using the SRB assay (Sigma–Aldrich) as previously reported [33]. In brief, after each incubation period, the cell monolayers were fixed with 10% trichloroacetic acid and stained with SRB for 30 min. The excess dye was subsequently removed by repeated washing with 1% acetic acid, after which the protein-bound dye was dissolved in 10 mM Tris solution for optical density (OD) measurement at 564 nm. The number of living cells was proportional to the amount of solubilized dye. Cell proliferation (%) was calculated as [(untreated—treated)/(untreated)] × 100 and plotted using GraphPad Prism 5.02.

### Multiplex immunostaining and immunolocalization analyses

Formalin-fixed paraffin-embedded (FFPE) tissue samples from human HER2 + BC patients who did or did not achieve pCR [pCR Yes (*n* = 18) and pCR No (*n* = 18), respectively] after neoadjuvant therapy were obtained from the archive of the INT. After deparaffinization and rehydration, an Opal 3-Plex Detection Kit was used (Cat. # NEL810001KT, Akoya Biosciences, Marlborough, MA, USA). Antigen retrieval was carried out in pH 9 buffer (cod. RE7119, Leica Biosystems, Buccinasco, Milan, Italy) by initial boiling at 100% power, followed by 20% power for 15 min, using microwave technology. The sections were treated with blocking buffer for 10 min at room temperature before primary antibody incubation. The slides were then incubated with a polymeric horseradish peroxidase (HRP)‐conjugated secondary antibody for 10 min, and signals were visualized using Opal 520 fluorophore‐conjugated tyramide signal amplification (TSA) reagent at a 1:100 dilution. Polymeric HRP catalyzes the covalent deposition of fluorophores around the marker of interest. The slides were again subjected to microwave treatment to strip the primary/secondary antibody complexes to allow staining of the next antigen with the corresponding antibody. The second round of staining was performed by incubation with the second primary antibody followed by incubation with the polymeric HRP-conjugated secondary antibody, incubation with Opal 620 fluorophore‐conjugated TSA reagent at a 1:100 dilution for signal visualization, and microwaving in antigen retrieval buffer. The following primary antibodies were used: mouse anti-human CD44v6 (clone VFF18, 1:500, cod. AB78960, Abcam, Cambridge, UK), rabbit anti-human ALDH1A1 (1:500, cod. GTX123973, GeneTex, Irvine, CA, USA), and rabbit anti-human CD36 (clone D8L9T, 1:200, cod. #14,347, Cell Signaling Technology, Danvers, MA, USA). For combined immunofluorescence and HER2 immunohistochemistry (IHC) analysis, after neutralization of endogenous peroxidase with 3% H_2_O_2_ and Fc blocking with 0.4% casein in phosphate buffer solution (PBS; Novocastra, Newcastle upon Tyne, UK), the same sections were incubated with a rabbit anti-HER2/ErbB2 antibody (clone D8F12, 1:250, cod. #4290, Cell Signaling Technology), followed by the components of a Novolink Polymer Detection System (Novocastra) and 3,3’-diaminobenzidine (DAB; Novocastra) as the substrate chromogen. Nuclei were counterstained with 4',6-diamidino-2-phenylindole (DAPI) and Harris hematoxylin (Novocastra). Staining was analyzed under a Zeiss Axioscope A1 microscope equipped with four fluorescence channels for widefield IF visualization. Micrographs were acquired using a Zeiss Axiocam 503 color digital camera connected to Zen 2.0 software (Zeiss, Oberkochen, Germany). Quantitative analysis of staining for multiple markers was performed via HALO image analysis software (v3.2.1851.229; Indica Labs, Albuquerque, NM, USA) and by evaluating the percentage of costaining in five nonoverlapping fields at medium magnification (200 ×).

### Mammosphere forming efficiency (MFE) assay

Dissociated cells were seeded in 6-well ultralow attachment plates (Corning, NY, USA) at a density of 2,500–3,000 cells per well in serum-free MammoCult medium (StemCell Technologies, Vancouver, Canada) and incubated for 7 days as previously reported [34]. In brief, engineered cell lines (MDAMB361 shSCR, shCD36 (1), shCD36 (2), HCC1954-Empty Vector and HCC1954-CD36 +) were treated on day 0 with 0.5 µM lapatinib or 0.1% DMSO. Similarly, HCC1954, MDAMB361, EFM192A and HCC1569 cells were treated on day 0 with 0.5 µM lapatinib and the anti-CD36 inhibitor SSO (50 µM) alone or in combination. The effect of CD36-silencing on the MFE (%) was also evaluated in EFM192A and HCC1569 transiently transfected with siCD36 and siSCR, as internal control, for 72 h and followed by in vitro culture under mammoshere formation-promoting conditions. The mammospheres (MS) were counted microscopically on day 7, and representative images were acquired using an EVOS XL Core Cell Imaging System (Thermo Fisher Scientific) (10 × magnification). The mammosphere forming efficiency (MFE) was calculated as the number of MSs divided by the number of single cells that were initially seeded, and MFE values are expressed as % means (± SDs). The MFE inhibition percentage, i.e., the decrease in the capability to form MSs under 3D culture conditions, was calculated as [(untreated MFE-treated MFE)/(untreated MFE)] × 100, as reported previously [34].

### Quantitative real-time PCR (qRT‒PCR)

Total RNA from the tested cells was extracted using QIAzol (QIAGEN) according to the manufacturer’s instructions and as reported previously [35], and RNA purity was verified by measuring the 260/280 absorbance ratio. cDNA was reverse transcribed from 1 µg of total RNA in a 20 µl reaction volume with SuperScript III (Invitrogen, Waltham, MA, USA) using oligo(dT) primers, and expression was measured by qRT‒PCR using an Applied Biosystems SYBR® Green dye-based PCR assay in an ABI Prism 7900HT sequence detection system (Applied Biosystems, Foster City, CA, USA). Human *CD36* and catenin beta 1 (*CTNNB1)* transcripts were amplified using the following primer pairs (200 nM each primer): *CD36* Forward: TCATGTCTTGCTGTTGATTTGTGA, *CD36* Reverse: TGGTTTCTACAAGCTCTGGTTCTTA; *CTNNB1* Forward: GTGCTATCTGTCTGCTCTAGTA, *CTNNB1* Reverse: CTTCCTGTTTAGTTGCAGCATC. All the experiments were performed in triplicate. Target gene expression data were normalized to actin beta expression data (*ACTB* Forward: AGGCATCCTCACCCTGAAG; *ACTB* Reverse: TCCATGTCGTCCCAGTTGGT). Gene expression analysis was performed using the comparative 2Δ (Ct) method with the housekeeping gene *ACTB* for normalization.

### ALDH activity assay

ALDH activity was measured in the tested cells using an ALDEFLUOR assay kit (StemCell Technologies) following the manufacturer’s instructions. In brief, 7.5 × 10^5^ cells were suspended in ALDEFLUOR assay buffer containing the ALDH substrate BODIPY-aminoacetaldehyde (BAAA) and incubated for 45 min at 37 °C. A specific inhibitor of ALDH, diethylaminobenzaldehyde (DEAB), was used to distinguish the ALDH + and ALDH– cell subsets.

### Vector construction, lentiviral particle production and tumor cell infection

The lentiviral vector encoding human CD36 was constructed using a third-generation self-inactivating lentiviral system that, based on four different plasmids, offers maximal biosafety. The backbone used was pRRL-sin-cPPt.CMV-GFP.WPRE (kindly provided by Dr. G. Ferrari, San Raffaele Scientific Institute, Milan), in which the GFP sequence was replaced with the cDNA sequence of human CD36. The cDNA sequence of CD36 was excised from pUC19-CD36 (GenScript, Piscataway, NJ, USA) using the restriction enzymes AgeI and SalI and was then ligated into the viral backbone and verified by sequencing. For CD36 silencing, pLKO.1-puro vectors carrying different short hairpin RNA (shRNA) sequences (TRCN0000057000; TRCN00000437740; TRCN00000419016) against CD36 were purchased from Sigma–Aldrich, along with a control nontargeting shRNA vector (SHC005). Lentiviral particles were produced using standard methods. In brief, lentiviral particles were produced by transient cotransfection of the transfer vector constructs (for either expression or silencing of CD36) with the VSV-G-expressing plasmid pMD.G and the third-generation packaging plasmids pMDLg/pRRE and pRSVRev [36] into 293 T cells. The medium was replaced 20 h later with fresh medium. The supernatant was collected 24 h later and used for target cell infection at a 1:2 concentration ratio in the presence of 8 µg/ml polybrene to facilitate viral entry into the cells. HCC1954 cells were infected with CD36-overexpressing or empty vector lentiviral particles to obtain the HCC1954-CD36 + and HCC1954-Empty Vector cell lines, respectively. MDAMB361 cells were infected with CD36-silencing and scrambled lentiviral particles to obtain the MDAMB361-shCD36 and MDAMB361-shSCR cell lines, respectively. The medium was changed the next day, and after 4 days, the transduction efficiency was evaluated by FACS analysis using an anti-CD36 antibody. For CD36 silencing, transduced cells were selected based on puromycin resistance, and CD36 downregulation was evaluated by flow cytometry.

### Transient silencing of CD36 and CTNNB1

For transient silencing of *CD36*, EFM192A and HCC1569 cells at 75–80% confluence were transfected with specified amounts of CD36 small interference RNA (siRNA) (100 nM; Assay ID: 105938) with Lipofectamine® RNAiMAX Reagent (Thermo Fisher Scientific) according to the manufacturer’s protocol. Cells were also transfected with Silencer™ Negative Control No. 1 siRNA (100 nM) (Thermo Fisher Scientific) following the same protocol for use as negative control cells. After 48 h, the transfected cells were tested for CD36 expression at both the transcriptional and translational levels. For transient silencing of *CTNNB1*, HCC1954, MDAMB361, EFM192A and HCC1569 cells at 75–80% confluence were transfected with specified amounts of *CTNNB1* siRNA (100 nM; Assay ID: 146154) with Lipofectamine® RNAiMAX Reagent (Thermo Fisher Scientific) according to the manufacturer’s protocol. Cells were also transfected with Silencer™ Negative Control No. 1 siRNA (100 nM) following the same protocol for use as negative control cells. After 24 h and 48 h, the transfected cells were tested for CD36 expression at both the transcriptional and translational levels.

### Western blot analysis

Protein extracts from HCC1954, MDAMB361, EFM192A, HCC1569, CD36 transiently silenced EFM192A and HCC1569, MDAMB361-shSCR and MDAMB361-shCD36 tumor cells were obtained by incubation for 40 min at 0 °C with RIPA Lysis and Extraction Buffer (Thermo Fisher Scientific) in the presence of Halt™ Protease Inhibitor Cocktail (Thermo Fisher Scientific), mixed with gel sample buffer under reducing conditions, and heated for 5 min at 95 °C. Extracted proteins were separated by electrophoresis on precast 4–12% Bis–Tris gels (Invitrogen, Thermo Fisher Scientific). The separated proteins were electrophoretically transferred onto nitrocellulose membranes, which were stained with Ponceau Red to assess protein loading, washed extensively with TBS + 0.5% Tween 20, and saturated for 1 h at room temperature in blocking solution (5% low-fat milk in TBS + 0.1% Tween 20). Primary antibodies in 3% low-fat milk in TBS + 0.1% Tween 20 were then added, and the membranes were incubated for 1 h at room temperature or overnight at 4 °C with gentle shaking. The following mouse monoclonal primary antibodies were used: anti-β-actin-peroxidase (AC-15 clone; 1:50.000; Sigma‒Aldrich) and anti-c-ErbB2/c-Neu (3b-5 clone; 1:300; Calbiochem, Darmstadt, Germany). The following rabbit monoclonal primary antibodies were used: anti-phospho-HER2/ErbB2 (Tyr1221/1222) (6B12 clone; 1:1000; Cell Signaling Technology) and anti-catenin beta 1 (1:1000; Cell Signaling Technology). The membranes were then washed extensively with TBS + 0.5% Tween 20 and incubated with sheep anti-mouse Ig (1:5.000) (Amersham GE Healthcare, Little Chalfont, UK) or with donkey anti-rabbit Ig (1:10.000; Amersham GE Healthcare) for 1 h at room temperature. Signals were detected using enhanced chemiluminescence (ECL; Amersham GE Healthcare).

### In vivo tumorigenicity assay

Mice were maintained under pathogen-free conditions at the animal facility of INT. All procedures were performed with ethical approval as stated in the Declarations (Ethical approval). Established and randomized 8-week-old female severe combined immunodeficient mice (SCID) mice were purchased from Charles River Laboratories (Calco, Italy). Mice (*n* = 5–6) were injected in a mammary fat pad (m.f.p.) with 9 × 10^5^ engineered lentivirally transduced MDAMB361 cells (MDAMB361-shCD36 or MDAMB361shSCR) pretreated in vitro for 72 h with the anti-HER2 inhibitor lapatinib or diluted DMSO as an internal control. Tumor size was evaluated once weekly by caliper measurement, and the approximate volume of each mass was calculated using the formula 0.5 x d1^2^ x d2, where d1 and d2 are the smaller and larger diameters, respectively. Mice were sacrificed when the tumor volume reached ~ 1000 mm^3^.

### Statistical analysis

Statistical analyses were conducted with GraphPad Prism 5.02 software using unpaired or paired two-tailed Student’s t test as appropriate. When *p* < 0.05, the difference between the compared groups was considered significant. The data are presented as the mean ± standard error of the mean (SEM) values (n ≥ 3 technical replicates). The sample size used in each individual experiment is reported in the corresponding figure legend. Mouse survival was assessed using the Kaplan–Meier method. Linear regression analysis was performed and the Pearson correlation coefficient (*r)* was calculated to estimate the correlations between 1) the percentages of HER2 + /CD44v6 + cells and HER2 + /CD36 + cells and 2) the percentages of ALDH1A + cells and CD36 + cells in primary HER2 + diagnostic core biopsies obtained from human patients.

## Results

### CD36 expression is linked to resistance to anti-HER2 therapy and to FA uptake in HER2 + BC patients and cells

**Fig. 1:**
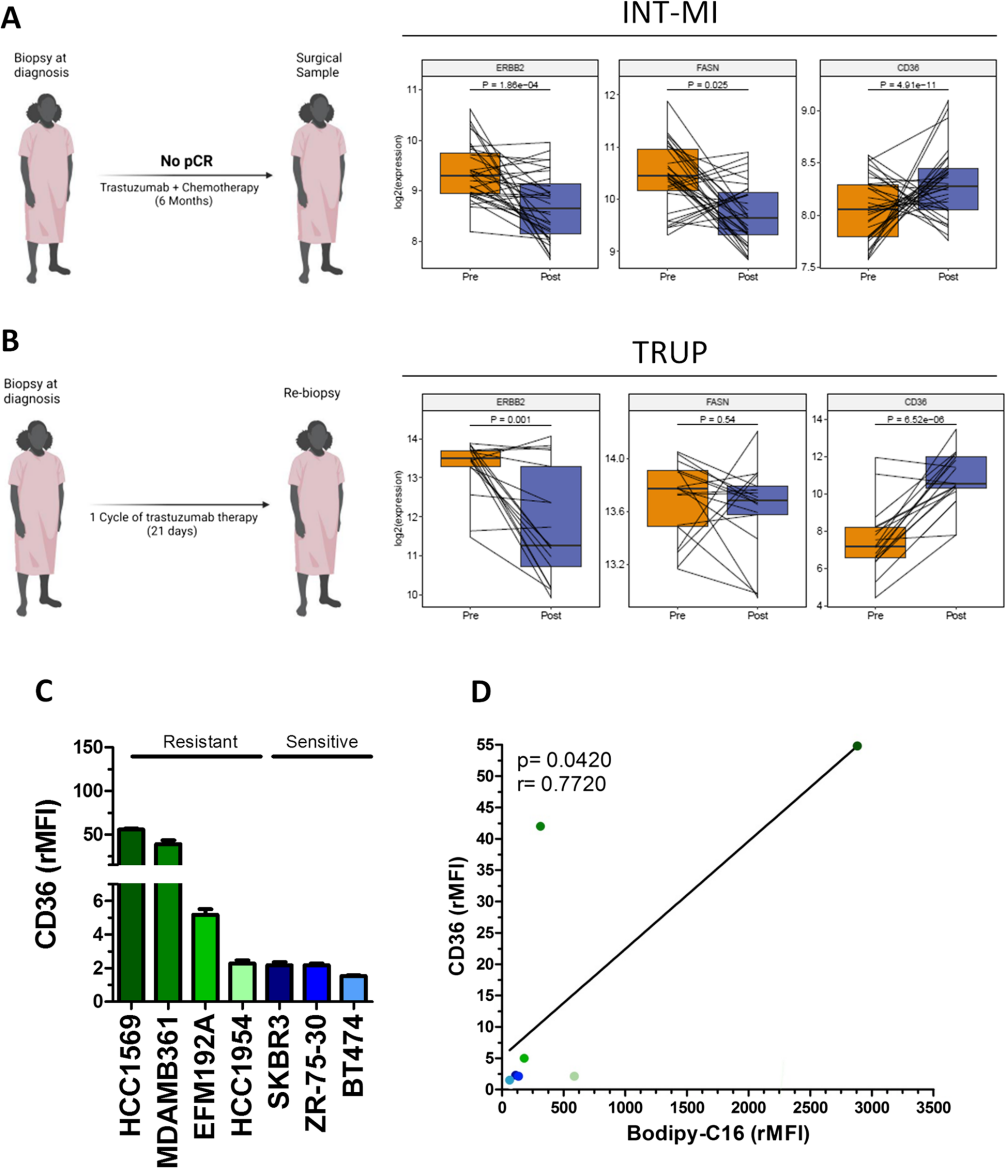
CD36 upregulation is a marker of resistance to anti-HER2 therapy in HER2 + BC. **A-B**, (Left) schedule of treatment administration for HER2 + BC patients in the INT-MI series (**A**) and the TRUP trial (**B**); (right) *ERBB2, FASN* and *CD36* transcript expression as determined by GEP in matched tumor specimens collected before and after neoadjuvant treatment in the INT-MI series (*n* = 32, A) and the TRUP trial (*n* = 17, B). Significance was calculated by two-tailed paired Student’s t test. **C**, rMFI of CD36 expression in anti-HER2 therapy-resistant (HCC1569, MDAMB361, EFM192-A, and HCC1954) and anti-HER2 therapy-sensitive (SKBR3, ZR7530, and BT474) HER2 + BC cell lines, as evaluated by FACS analysis. The data are presented as the means ± SEMs (*n* = 3). **D**, Pearson correlation analysis between the CD36 rMFI and BODIPY-C16 rMFI (%) in the above mentioned resistant/sensitive HER2 + BC cell lines

We first investigated the expression of *ERBB2*, *FASN* and *CD36* transcripts in matched diagnostic and surgical samples derived from the INT-MI series (Table 1). In comparing the pretherapy *versus (vs.)* the posttherapy samples (*n* = 32), we observed a significant decrease in *ERBB2* and *FASN* expression, as expected, and a significant increase in *CD36* expression (Fig. 1A) in the posttherapy samples, suggesting that trastuzumab-resistant tumor cells could undergo a switch in their FA source from FASN-mediated de novo biosynthesis to FA uptake from the extracellular environment via the reprogramming of CD36 expression/activity [37]. To validate the INT-MI series molecular data and assess whether HER2 inhibition by trastuzumab affects *ERBB2*, *FASN,* and *CD36* transcript levels, we analyzed a gene expression dataset of matched core biopsies (*n* = 17) collected from HER2 + BC patients enrolled in the TRUP window-of-opportunity trial before and after one cycle of trastuzumab monotherapy (Fig. 1B) [31]. Consistent with the above results, *CD36* expression was significantly increased following trastuzumab treatment, accompanied by a significant decrease in *ERBB2* expression, in post-trastuzumab patients in the TRUP trial (Fig. 1B), confirming the ability of anti-HER2 therapy to upregulate FA uptake in HER2 + BC cells. To evaluate whether CD36 upregulation was linked to changes in FA uptake and lipid metabolism, we stratified patients of the INT-MI series into two groups: those with increased CD36 expression (ΔCD36 + ; *n* = 20) and those with decreased expression (ΔCD36 − ; *n* = 12) following trastuzumab-based neoadjuvant therapy (Table 1). Of note, FA-related pathways, including hallmark adipogenesis, cholesterol homeostasis, and fatty acid metabolism, were positively enriched post-treatment exclusively in tumors with ΔCD36 + but not in those ΔCD36 − (Supplementary Fig. S1A). Furthermore, analysis of lipid metabolic pathways revealed significant enrichment of gene sets associated with cellular lipid responses and lipid metabolism exclusively in ΔCD36 + tumors (Supplementary Fig. S1B).

To investigate whether the CD36 expression level is also associated with resistance to anti-HER2 therapy in the preclinical setting, CD36 protein expression was evaluated by flow cytometry in a panel of HER2 + BC cell lines with different susceptibility to anti-HER2 drugs (Fig. 1C), which were previously characterized for their sensitivity to the HER2 tyrosine kinase inhibitor (TKI) lapatinib ( Supplementary Fig. S2). Consistent with the above findings, the CD36 level was greater in lapatinib-resistant HCC1569, MDAMB361, and EFM192A cells than in lapatinib-sensitive SKBR3, ZR75.30, and BT474 cells (Fig. 1C; Supplementary Fig. S2). We then tested FA internalization in the above mentioned target cells via flow cytometry using BODIPY-FL C_16_, a fluorescently tagged form of palmitic acid. We observed a direct significant correlation between the CD36 expression level and FA uptake capability, confirming the greater FA internalization capacity of resistant HER2 + BC cells than sensitive HER2 + BC cells (Fig. 1D). Taken together, these findings link CD36 expression with a greater capacity to internalize FAs.

### CD36 expression is enriched in the HER2 + EMT-like BCSC subset

**Fig. 2:**
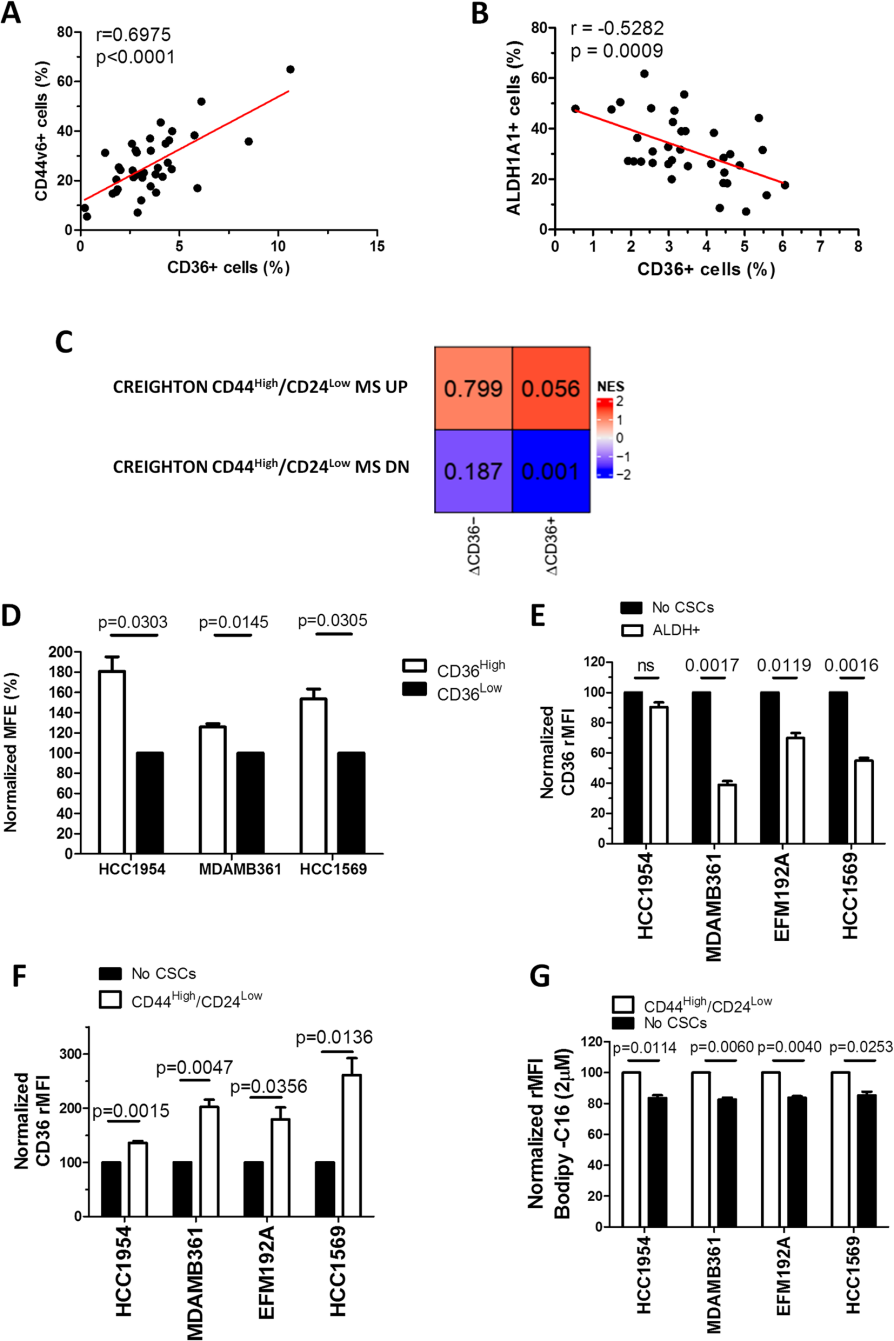
CD36 expression is enriched in EMT-like CSCs of the HER2 + BC cell population. **A-B**, linear regression analysis of the percentages of HER2 + /CD36 + compared with HER2 + /CD44v6 + (**A**) and with HER2 + /ALDH1A1 + (**B**) atypical malignant stem-like cells in 36 human primary HER2 + diagnostic core biopsies obtained from BC patients in the pCR Yes and pCR No groups and subjected to multiplex immunostaining and visualization by widefield microscopy. **C**, normalized enrichment scores (NESs) and *p* values of the upregulated Creighton CD44^High^/CD24^Low^ MS signature (UP) and downregulated Creighton CD44^High^/CD24^Low^ MS signature (DN) in the INT-MI dataset stratified according to increased (ΔCD36 +) or decreased (ΔCD36-) CD36 expression in post- *vs.* pre-neoadjuvant therapy biopsies. **D**, MFE (%) of HCC1954, MDAMB361 and HCC1569 cells sorted according to the CD36 level (CD36^High^ *vs.* CD36^Low^). The data are presented as the means ± SEMs (*n* = 3); significance was calculated using two-tailed paired Student’s t test. **E–F**, normalized CD36 rMFI in the ALDH + (**E**) and CD44^High^/CD24^Low^ (**F**) CSC subsets *vs.* the corresponding No CSCs subsets of HCC1954, MDAMB361, EFM192A and HCC1569 cells, as evaluated by FACS analysis. The data were normalized to the CD36 rMFI in the No CSCs subset. The data are presented as the means ± SEMs (*n* = 3); significance was calculated using two-tailed paired Student’s t test. **G**, normalized BODIPY-C16 rMFI in the CD44^High^/CD24^Low^ CSC subsets *vs.* the corresponding No CSCs subsets of HCC1954, MDAMB361, EFM192A and HCC1569 cells, as evaluated by FACS analysis. The values were normalized to the BODIPY-C16 rMFI in the No CSCs subset. The data are presented as the means ± SEMs (*n* = 3); significance was calculated using two-tailed paired Student’s t test

To address the role of CD36-positive CSCs in anticancer treatment resistance, we examined CD36 expression in mesenchymal EMT-like and epithelial MET-like [38, 39] BCSC subsets. To this end, diagnostic HER2 + BC biopsies from selected neoadjuvant-treated HER2 + BC patients who did or did not achieve pCR, identified as the pCR Yes (*n* = 18) and pCR No (*n* = 18) cases, respectively (Table 2), were subjected to in situ assessment of the percentage of HER2 + cells also expressing CD36 (CD36 + cells %) or CD44v6 (CD44v6 + cells %) (Fig. 2A), a specific CD44 isoform enriched in EMT-like stem cells [40]. Further, we performed an in situ assessment of the percentage of HER2 + cells also expressing CD36 (CD36+ cells %) or aldehyde dehydrogenase family 1 member A1 (ALDH1A1+ cells %) (Fig. 2B), a marker enriched in MET-like stem cells [41]. In situ analysis of the HER2 + /CD36 + and HER2 + /CD44v6 + cell percentages (Fig. 2A) and HER2+/CD36 + and HER2 +/ALDH1A1 + cells percentages (Fig. 2B) revealed that the CD36 + cell percentage was positively correlated with the proportion of mesenchymal neoplastic stem-like cells and negatively correlated with that of epithelial neoplastic stem-like cells, indicating that CD36 is prevalently expressed in EMT-like CSCs. To test the clinical relevance of CD36 enrichment in HER2 + EMT-like stem cells (Fig. 2A), we evaluated the enrichment of the Creighton CD44^High^CD24^Low^ MS stemness signature designed specifically to identify EMT-like CSCs [27] in posttherapy HER2 + BC samples *vs.* their matched baseline counterparts in ΔCD36 + and ΔCD36 − tumors by GSEA (Fig. 2C). The genes in the Creighton CSC signature that were upregulated (Creighton CD44^High^/CD24^Low^ MS UP) or downregulated (Creighton CD44^High^/CD24^Low^ MS DN) were significantly positively and negatively enriched, respectively, only in the ΔCD36 + subset (Fig. 2C). The enrichment was specific to EMT-like CSCs, as no comparable enrichment was observed with other published CSC signatures (Supplementary Fig. S3A), indicating intratumor enhancement of mesenchymal-like BCSCs in tumors that exhibited increased CD36 expression upon trastuzumab treatment. This increment resulted with a positive correlation between the Creighton signature and CD36 expression in HER2 + residual BC samples following treatment, while showing a negative correlation with FA biosynthesis and FASN (Supplementary Figure S3B).

Then, lapatinib-resistant HCC1954, MDAMB361 and HCC1569 cells were sorted according to the CD36 expression level (CD36^High^ *vs.* CD36^Low^) by flow cytometry (Supplementary Fig. S4). However, we failed to efficiently sort EFM192A lapatinib-resistant cells into CD36^High^ and CD36^Low^ subsets due to their high sensitivity to the physical forces exerted during cell sorting. As shown in Fig. 2D, the sorted CD36^High^ cells exhibited a significantly greater MFE, *i.e.,* an increased number of 3D multicellular structures enriched in BCSCs [42], than did the sorted CD36^Low^ cells across all the HER2 + BC cell lines. In this context, the significantly increased MFE (%) of the CD36^High^ HCC1954 cell subset supports the hypothesis that CD36 is expressed mainly in the stem cell component compared with the tumor bulk counterpart (No CSCs) of this cell line. Next, we investigated the differential expression of the CD36 protein in HCC1954, MDAMB361, EFM192A and HCC1569 MET- (ALDH +) and EMT-like (CD44^High^/CD24^Low^) BCSCs compared with the matched No CSCs components by FACS analysis. This evaluation revealed lower CD36 expression in HER2 + ALDH + BCSCs (Fig. 2E; Supplementary Fig. S5) and, conversely, higher CD36 expression in CD44^High^/CD24^Low^ HER2 + BCSCs (Fig. 2F; Supplementary Fig. S5B) than in the matched No CSCs counterpart. Additionally, we compared the ability of the CD44^High^/CD24^Low^ BCSC subsets to internalize FAs from culture supernatants with that of the matched No CSCs subsets using the BODIPY-FL C16 assay. As shown in Fig. 2G, the gated CD44^High^/CD24^Low^ HER2 + BCSCs exhibited increased FA uptake compared with that of their matched No CSCs counterparts (Fig. 2G; Supplementary Fig. S5C). Overall, our findings support the candidacy of CD36 as a metabolic marker and potential therapeutic target for mesenchymal HER2 + CSCs.

### High CD36 expression/activity promotes an EMT-like CSC state

**Fig. 3:**
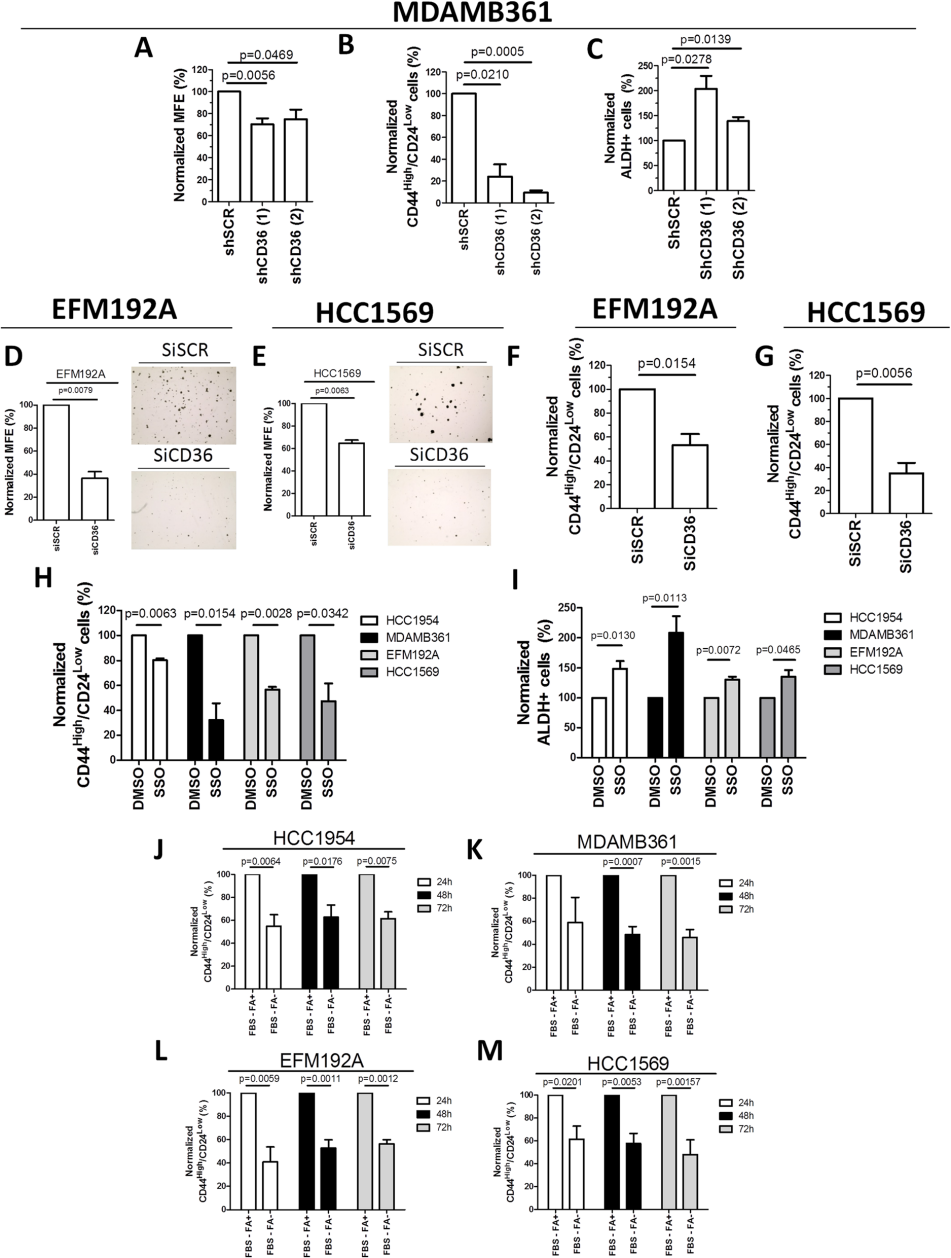
CD36 expression/activity promotes the maintenance of an EMT-like CSC phenotype. **A**, normalized MFE (%) of engineered MDAMB361-shSCR, MDAMB361-shCD36 (1) and MDAMB361-shCD36 (2) cells cultured under MS-promoting conditions for 7 days. The data were normalized to the MFE (%) of MDAMB361-shSCR cells. The data are presented as the means ± SEMs (*n* = 5); significance was calculated using two-tailed paired Student’s t test. **B-C**, summary of data showing the percentages of CD44^High^/CD24^Low^ (**B**) and ALDH + (**C**) CSCs among engineered MDAMB361-shSCR, MDAMB361-shCD36 (1) and MDAMB361-shCD36 (2) cells, as evaluated by FACS analysis. The values were normalized to the percentage of CSCs among MDAMB361-shSCR cells. The data are presented as the means ± SEMs (*n* = 3 in **B** and *n* = 5 in **C**); significance was calculated using two-tailed paired Student’s t test. **D-E**, Normalized MFEs (%) (Left) and representative MS pictures (Right) of EFM192A (**D**) and HCC1569 cells (**E**) transiently transfected with scrambled siRNA (siSCR) or CD36-targeting siRNA (siCD36) for 72 h. After transfection, the cells were cultured under MS-promoting conditions for 7 days. The data were normalized to the MFE (%) of EFM192A- and HCC1569-siSCR cells. The data are presented as the means ± SEMs (*n* = 3); significance was calculated using two-tailed paired Student’s t test. **F-G**, summary of data showing the percentages of CD44^High^/CD24^Low^ CSCs among transiently transfected EFM192A-siSCR and EFM192A-siCD36 cells (**F**) and HCC1569-siSCR and HCC1569-siCD36 cells (**G**), as evaluated by FACS analysis. The values were normalized to the percentage of CD44^High^/CD24^Low^ CSCs among siSCR cells. The data are presented as the means ± SEMs (*n* = 4); significance was calculated using two-tailed paired Student’s t test. **H-I**, summary of data showing the percentages of CD44^High^/CD24^Low^ CSCs (**H**) and ALDH + CSCs (**I**) among HCC1954, MDAMB361, EFM192A and HCC1569 cells treated with DMSO or SSO (100 μM) for 48 h, as evaluated by FACS analysis. The values were normalized to the percentage of CSCs among DMSO-treated cells. The data are presented as the means ± SEMs (*n* = 4); significance was calculated using two-tailed paired Student’s t test. **J-M** summary of data showing the percentages of CD44^High^/CD24^Low^ CSCs in HCC1954 (**J**), MDAMB361 (**K**), EFM192A (**L**) and HCC1569 (**M**) cells cultured in media supplemented with FBS depleted (FBS-FA-) or not (FBS-FA +) of FA for 24 h, 48 h and 72 h. The values were normalized to the percentage of CSCs in FBS-FA + cultured cells. The data are presented as the means ± SEMs (*n* = 5–6); significance was calculated using two-tailed paired Student’s t test

To investigate the potential role of CD36 in the maintenance of CD44^High^/CD24^Low^ CSCs, we stably silenced CD36 expression in MDAMB361 cells using two different shRNAs [shCD36 (1) and shCD36 (2)]. The same cells were transduced with a scrambled shRNA (MDAMB361-shSCR) as an internal control (Supplementary Fig. S6A). Notably, while HER2 protein and activation (pHER2) remain similar (Supplementary Fig. S6B), the MFEs (%) of MDAMB361-shCD36 (1) and MDAMB361-shCD36 (2) cells were significantly lower than that of MDAMB361-shSCR cells (Fig. 3A). indicating an active role for CD36 in HER2 + BCSC proliferation/enrichment. Similarly, compared with control MDAMB361-shSCR cells, the MDAMB361-shCD36 (1) and MDAMB361-shCD36 (2) cells exhibited a significantly lower percentage of EMT-like CSCs (Fig. 3B and Supplementary Fig S7A). Conversely, the percentage of ALDH + cells was significantly greater in the MDAMB361-shCD36 (1) and MDAMB361-shCD36 (2) cell populations than in the counterpart shSCR population (Fig. 3C; Supplementary Fig. S7B and C). Notably, we observed that MDAMB361-shSCR cells maintained a higher percentage of CD44^High^/CD24^Low^ CSCs compared to MDAMB361-shCD36 (1) and MDAMB361-shCD36 (2) cells, even when cultured under 3D conditions (Supplementary Fig. S8). All these data suggest that CD36 could play an important role in CSC plasticity via the induction of an EMT-like state. To strengthen these findings, EFM192A and HCC1569 cells were transiently transfected with a siRNA designed to impair CD36 expression (siCD36) and, in parallel, with an internal control siRNA (siSCR) (Supplementary Fig. S9A). As observed for MDAMB361 stably silenced for CD36 expression, the transient inhibition of CD36 expression in the EFM192A and HCC1569 cell lines did not alter the expression/activation of HER2 compared with the corresponding internal experimental controls (siSCR) (Supplementary Fig. S9B). Notably, EFM192A and HCC1569 cells transiently silenced for CD36 (siCD36), showed a remarkable decrease of MFE (%) (Fig. 3D and E) and of the CD44^High^/CD24^Low^ cell population (Fig. 3F and G and Supplementary Fig. S10) vs. matched scramble siRNA (siSCR) cell counterparts, despite similar basal HER2 expression/activation (Supplementary Fig. S9B).

To investigate whether the inhibition of CD36 activity, *i.e.,* the decrease in the ability to uptake FA, in lapatinib-resistant cells can affect the percentages of CD44^High^/CD24^Low^ and ALDH + cells, we treated HCC1954, MDAMB361, EFM192A, and HCC1569 cells for 72 h with a nontoxic concentration (100 µM) of SSO (Supplementary Fig. S11), an irreversible inhibitor of FA internalization via the CD36 translocase. Inhibition of CD36 activity significantly decreased the percentage of EMT-like CSCs (Fig. 3H and Supplementary Fig. S12A) and, conversely, induced a marked increase in the enzymatic activity of ALDH, resulting in an increased number of ALDH + cells in all the tested cell lines (Fig. 3I; Supplementary Fig. S12B and C). Taken together, these data support the potential implication of CD36 expression/activity and FA uptake in the CSC formation and transition from an epithelial to a mesenchymal state. To further support this speculation, the human HER2 + BC cell lines HCC1569, MDAMB361, EFM192A and HCC1954, which have different susceptibilities to lapatinib treatment (Supplementary Figure S2), were grown in vitro in monolayer in the presence of fetal bovine serum (FBS) containing FAs (FBS—FA +) or not (FBS—FA-) for 24 h, 48 h and 72 h, and then the percentage of CD44^High^/CD24^Low^ cells was assessed via flow cytometry. As shown, the depletion of FA from FBS (FBS-FA-) added to any distinct cell culture medium significantly decreased the formation/number of EMT-like CD44^High^/CD24^Low^ cells compared with that generated in the matched cells cultured in vitro with the addition of FA to FBS (FBS-FA +) Fig. 3J-M and Supplementary Fig. S13). Further, HCC1954 cells with low CD36 expression were transduced with a lentiviral vector to stably induce CD36 overexpression (HCC1954-CD36 + cells) or with an empty vector as a control (HCC1954-Empty vector cells) (Supplementary Fig. S14). Notably, ectopic CD36 overexpression increased both the MFE (%) of the HCC1954-CD36 + cells (Supplementary Fig. S15A) and the percentage of CD44^High^/CD24^Low^ CSCs among these cells (Supplementary Fig. S15B) compared with those of their control counterparts. Taken together, these results identify CD36 as a functional metabolic hub for enriching EMT-like CSCs in HER2 + BC.

### The Wnt pathway regulates CD36 expression in HER2 + BC cells

**Fig. 4:**
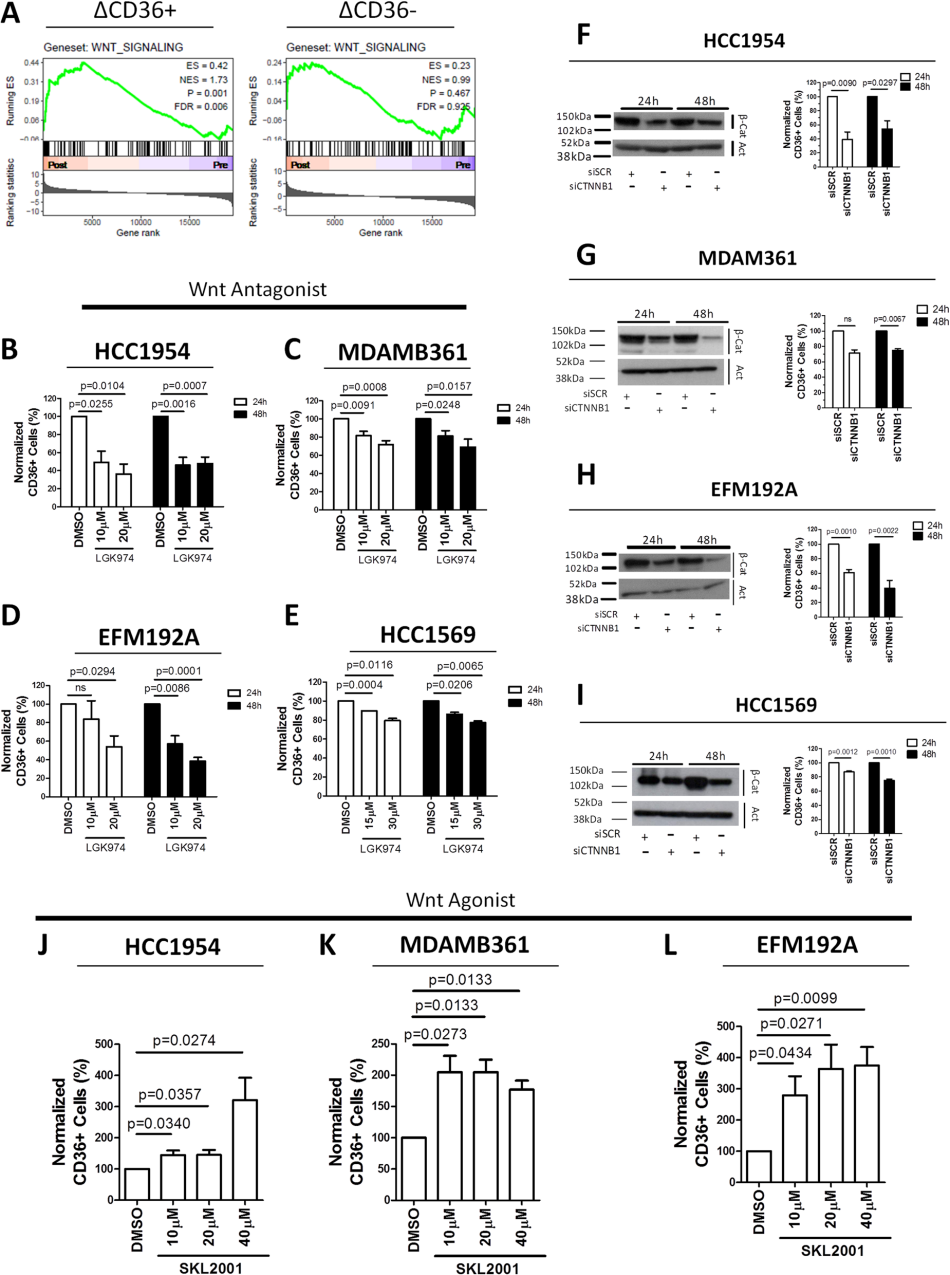
The Wnt pathway regulates the expression of CD36 in HER2 + BC cells. **A**, GSEA enrichment plots of Wnt gene sets (KEGG) in post- *vs.* pretreatment samples from patients in the INT-MI series based on increased (ΔCD36 + , left) or decreased (ΔCD36-, right) CD36 expression. NES: normalized enrichment score; FDR: false discovery rate. **B-E**, percentages of CD36 + cells among HCC1954 (**B**), MDAMB361 (**C**), EFM192A (**D**) and HCC1569 (**E**) cells after treatment for 24 h or 48 h with the WNT inhibitor LGK974 or the diluent DMSO. The data were normalized to the percentages of CD36 + cells among the corresponding DMSO-treated cells. The data are presented as the means ± SEMs (*n* = 4–7); significance was calculated using two-tailed paired Student’s t test. **F-I**, (left) catenin beta 1 expression (ß-Cat) as evaluated by Western blotting in HCC1954 (**F**), MDAMB361 (**G**), EFM192A (**H**) and HCC1569 (**I**) cells with transient silencing of *CTNNB1* (siCTNNB1) or transfected with the nonspecific siRNA construct (siSCR) for 24 h and 48 h. Actin was used to normalize protein loading; (right) percentages of CD36 + cells among HCC1954 (**F**), MDAMB361 (**G**), EFM192A (**H**) and HCC1569 (**I**) cells with or without (siSCR) transient silencing of catenin beta 1 (siCTNNB1) for 24 h and 48 h. The values were normalized to the percentage of CD36 + cells among siSCR-transfected cells. The data are presented as the means ± SEMs (*n* = 3–4); significance was calculated using two-tailed paired Student’s t test. **J-L**, percentages of CD36 + cells among HCC1954 (**J**), MDAMB361 (**K**) and EFM192A (**L**) cells treated for 24 h with the specific WNT agonist SKL2001 (10 μM, 20 μM or 40 μM) or the diluent DMSO. The values were normalized to the percentage of CD36 + cells among DMSO-treated cells. The data are presented as the means ± SEMs (*n* = 4–6); significance was calculated using two-tailed paired Student’s t test

To investigate the molecular mechanism involved in CD36 expression in EMT-like HER2 + CSCs, we tested the enrichment of the CSC-related gene signaling pathways, *i.e.,* the Wnt, TGF-beta, Hedgehog, NF-kB, JAK/STAT and Notch, which regulate stem cell activities [26, 43, 44], in post- *vs*. pretreatment cases in the INT-MI series based on increased (ΔCD36 +) or decreased (ΔCD36-) CD36 expression. Only the Wnt pathway was significantly enriched following trastuzumab-containing therapy, and this was specifically observed in HER2 + ΔCD36 + BC (Fig. 4A and Supplementary Fig. S16). These molecular findings support the existence of a link between the Wnt transcriptional regulatory network and CD36 expression. To assess whether pharmacological modulation of the Wnt pathway can affect CD36 expression, we treated HCC1954, MDAMB361, EFM192A and HCC1569 cells for 24 h and 48 h with LGK974 (Supplementary Fig. S17; Fig. 4B-E), a potent and specific Porcupine (PORCN) inhibitor [45] tested in the clinical setting (NCT01351103). Specifically, HCC1954, MDAMB361 and EFM192A cells were incubated with 10 or 20 μM LGK974 (Fig. 4B-D), whereas HCC1569 cells were incubated with 15 or 30 μM LGK974 (Fig. 4E) due to the very high expression of CD36 in these cells (Fig. 1C). Consistent with our hypothesis, LGK974 treatment significantly reduced CD36 transcript expression (Supplementary Fig. S18A-D) and the percentage of CD36 + cells in all the tested cell lines, albeit to different extents, compared with those in the DMSO treatment control groups, as assessed by FACS analysis (Fig. 4B-E; Supplementary Fig. S19). To further confirm the presence of a functional link between Wnt signaling and the percentage of HER2 + /CD36 + neoplastic cells, the same cell lines were transiently transfected with a specific siRNA designed to silence the *CTNNB1* gene, whose translated product acts as an intracellular signal transducer in the Wnt signaling pathway [44]. Notably, silencing of *CTNNB1*, as evaluated by Western blot (Fig. 4F-I, left) and qRT‒PCR (Supplementary Fig. S20A-D) analyses, induced a reduction in both *CD36* transcript expression (Supplementary Fig. S20A-D) and the percentage of CD36 + cells in all tested target cells, as evaluated by FACS analysis (Fig. 4F-I, right; Supplementary Fig. S21).

To further validate the interplay between Wnt signaling and CD36 expression, cells were treated with increasing nontoxic concentrations (10 µM, 20 µM, and 40 µM) of the Wnt agonist SKL2001 (Supplementary Fig. S22), a compound that mimics the effects of the Wnt ligand by disrupting the Axin/catenin beta 1 interaction and promoting catenin beta 1 translocation into the nucleus [46], or with DMSO as a control for 24 h (Fig. 4J-L).

Consistent with the above results, SKL2001 treatment tested at 10 µM, 20 µM, 40 µM increased the CD36 mRNA level (Supplementary Fig. S23) and the percentage of CD36 + cells in HCC1954, MDAMB361, and EFM192A cells (Fig. 4J-L; Supplementary Fig. S24). HCC1569 cells, which exhibit high intrinsic CD36 expression, were not treated with SKL2001. Overall, our results provide evidence of the tight interplay between Wnt signaling regulation/activation and CD36 expression in the CSC subset of HER2 + BC cells.

### Dual blockade of CD36 and HER2 increases the anti-CSC efficacy of anti-HER2 drugs

**Fig. 5:**
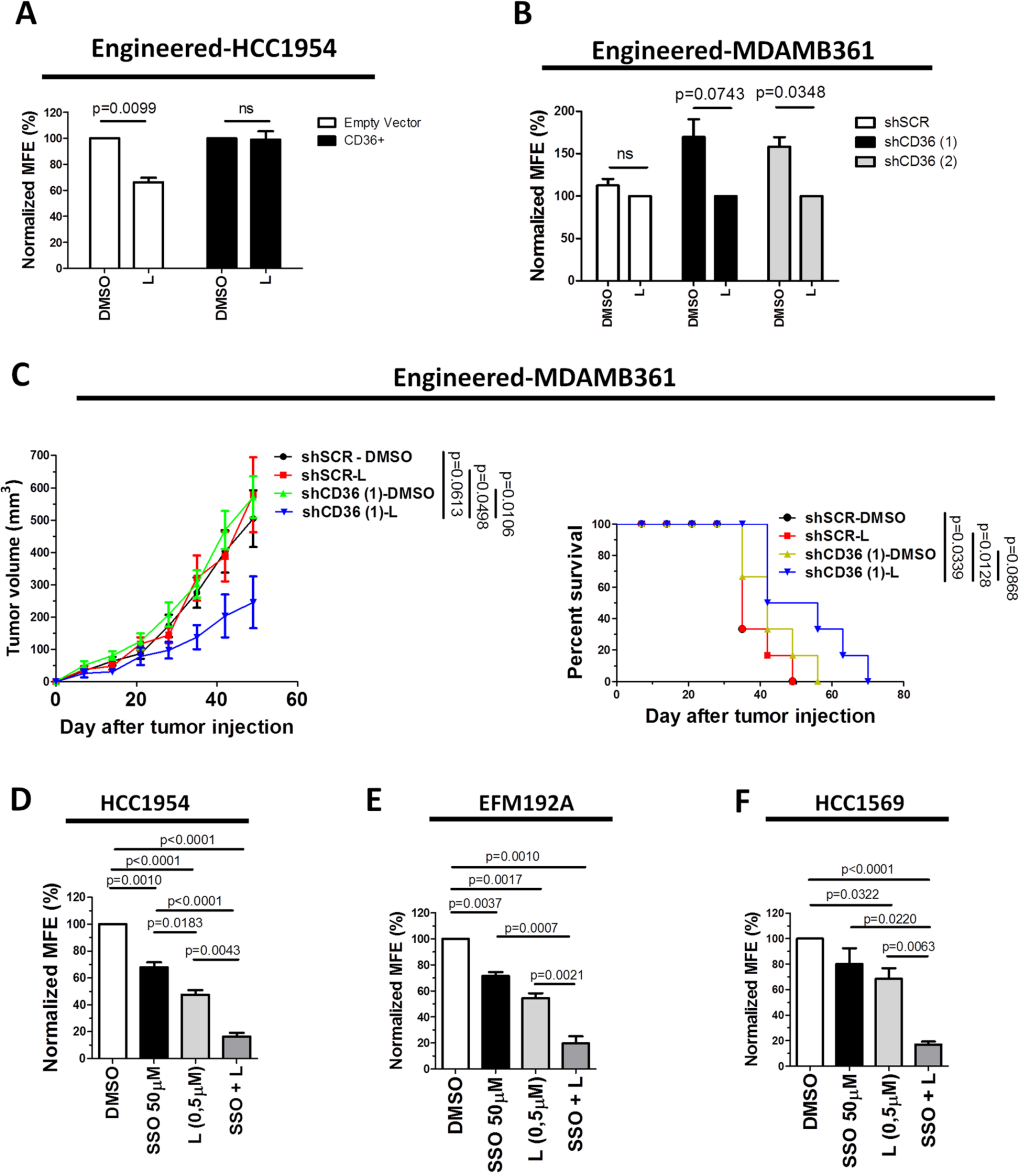
Dual blockade of CD36 and HER2 enhances the anti-CSC efficacy of anti-HER2 drugs in lapatinib-resistant HER2 + BC cells. **A-B**, normalized MFE (%) of HCC1954 cells transiently transduced with the CD36 overexpression plasmid (CD36 +) or empty vector (**A**) and of stably transduced MDAMB361-shSCR, MDAMB361-shCD36 (1) and MDAMB361-shCD36 (2) cells (**B**) treated with lapatinib (L) (0.5 μM) or DMSO. MSs were counted after 7 days of culture. The values were normalized to the MFE (%) of HCC1954-Empty Vector cells (**A**) or MDAMB361-shSCR cells (**B**). The data are presented as the means ± SEMs (*n* = 3); significance was calculated using two-tailed paired Student’s t test. **C**, in vivo tumor growth (left) and survival (right) were evaluated in SCID mice injected in a m.f.p. with 9 × 10^5^ engineered MDAMB361-shSCR or MDAMB361-shCD36 (1) cells pretreated in vitro with lapatinib (L, 0.45 μM) or DMSO for 72 h. (Left) The data are presented as the means ± SEMs (*n* = 5–6); significance was calculated using two-tailed unpaired Student’s t test. Mice were sacrificed when the tumor volume reached 750 mm^3^. (Right) Significance was determined by the log–rank (Mantel–Cox) test. **D-F**, normalized MFE (%) of HCC1954 (**D**), EFM192A (**E**) and HCC1569 (**F**) cells treated with SSO (50 μM) and lapatinib (L 0.5 μM) alone or in combination or with DMSO as an internal control. MSs were counted after 7 days of culture. The values were normalized to the MFE (%) of DMSO-treated cells. The data are presented as the means ± SEMs (*n* = 4); significance was calculated using two-tailed paired Student’s t test

To investigate whether an ectopic increase in CD36 expression can affect the MFE (%) of HCC1954 cells and modulate their susceptibility to anti-HER2 drugs, cells were transduced with the CD36 lentiviral vector (HCC1954-CD36 + cells) or empty vector (Empty Vector cells), as a control. The cells were subsequently cultured under MS-promoting conditions and treated with 0.5 μM lapatinib for 7 days (Fig. 5A). Notably, MSs derived from HCC1954-CD36 + cells were more refractory to lapatinib than were those derived from the counterpart Empty Vector cells (Fig. 5A). Conversely, to examine whether CD36 downregulation can impact sensitivity to lapatinib in MDAMB361 cells with stable CD36 silencing, we incubated MDAMB361-shCD36 (1), MDAMB361-shCD36 (2) and control MDAMB361-shSCR cells under MS-promoting conditions in either the presence or absence of lapatinib (0.5 μM). As shown in Fig. 5B, lapatinib treatment significantly decreased the MFE (%) of MDAMB361-shCD36 (1) and MDAMB361-shCD36 (2) cells compared to that of the DMSO control-treated cells, whereas MS formation by MDAMB361-shSCR cells was not affected by lapatinib treatment (Fig. 5B). Then, to evaluate whether dual blockade of CD36 and HER2 can affect the tumor-initiating cell component of HER2 + resistant BC cells in vivo, engineered MDAMB361-shSCR and MDAMB36-shCD36 (1) cells were pretreated in vitro for 72 h with 0.45 μM lapatinib or DMSO (as a control) and were then injected into a m.f.p. of SCID mice (*n* = 9 × 10^5^ cells/mouse). Significant inhibition of tumor growth was observed in mice after injection of lapatinib (L)-pretreated MDAMB361-shCD36 (1) cells compared with L-pretreated MDAMB361-shSCR cells or DMSO-pretreated MDAMB361-shCD36 (1) cells (Fig. 5C, left). Accordingly, the survival time in the group of mice injected with L-pretreated MDAMB361-shCD36 cells (1) was longer than that in either control animal group injected with DMSO- or L-pretreated MDAMB361-shSCR cells (Fig. 5C, right). Additionally, HCC1954, EFM192A and HCC1569 cells cultured under MS-promoting conditions were treated with SSO and a low dose (0.5 µM) of lapatinib alone or in combination. Notably, we found that compared with DMSO, both SSO and lapatinib inhibited MS formation in all the tested target cells, albeit to different extents, with significantly greater therapeutic activity when applied in combination (Fig. 5D-F). Taken together, these data suggest that concomitant treatment with CD36 and HER2 inhibitors may result in enhanced anti-CSC efficacy of anti-HER2 drugs in resistant HER2 + BC cell models.

### Expression of CD36 in intratumor HER2 + /CD44v6 + neoplastic stem-like cell subset is associated with resistance to trastuzumab in HER2 + BC patients

To assess whether the differential expression of CD36 in EMT-like *vs.* MET-like neoplastic stem-like cell subsets can be used to predict susceptibility to trastuzumab, we tested FFPE diagnostic biopsies obtained from HER2 + BC patients who received neoadjuvant trastuzumab plus chemotherapy (*n* = 36) by multiplex immunostaining (Table 2)*.*

**Fig. 6:**
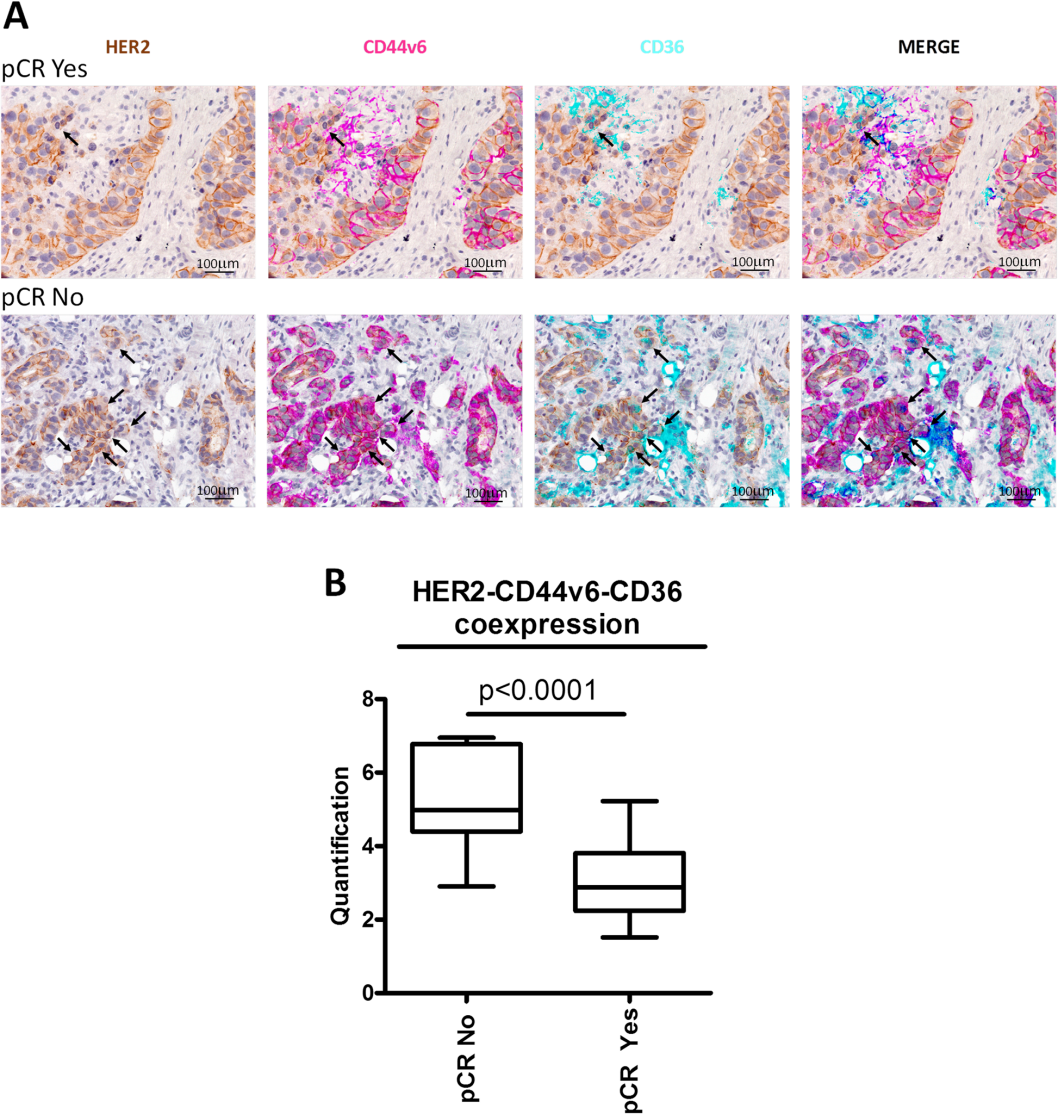
Enrichment of HER2 + /CD44v6 + /CD36 + stem-like cells is associated with resistance to therapy in HER2 + BC patients. **A**, representative micrographs of triple immunofluorescence staining for HER2 (brown signal), CD44v6 (pink signal) and CD36 (cyan signal) in pCR Yes and pCR No HER2 + BC biopsies. Coexpression of all three signals is indicated by the black arrows. Scale bars = 100 μm. **B**, quantification of the percentage of HER2 + /CD44v6 + /CD36 + atypical malignant stem-like cells in pCR Yes (*n* = 18) and pCR No (*n* = 18) HER2 + BC biopsies. Significance was calculated using two-tailed unpaired Student’s t test

To examine the coexpression of HER2, CD44v6, and CD36 in HER2 + BC patients with or without pCR, we combined immunofluorescence and IHC staining of FFPE tissue samples, with analysis through a digital quantitative pathology approach (see Materials and Methods). This approach allowed the evaluation of CD44v6/CD36 stem-like markers in association with routine HER2 IHC staining. The percentage of intratumor stem-like cells coexpressing the three markers was determined. As shown in Fig. 6A and B, a significantly higher frequency of CD44v6/CD36 + stem-like cells was found in biopsies from HER2 + BC patients who did not achieve pCR (pCR No patients) after neoadjuvant treatment with trastuzumab-based therapy than in biopsies from pCR Yes patients, providing striking clinical evidence corroborating our observations in all of our tested 2D and 3D HER2 + BC cell models and in mice.

## Discussion

Cancer metabolism is emerging as a crucial regulator of biological processes related to tumor cell features such as aggressiveness, metastatic capacity, immunoregulatory properties, and therapeutic resistance [47–49]. In particular, the reprogramming of CD36-mediated FA uptake in lipogenic HER2 + BC constitutes a mechanism of resistance to HER2-targeted drugs by fulfilling the requirement for lipids that is compromised by pharmacological inhibition of the HER2-FASN axis following treatment with HER2 and/or FASN inhibitors, as recently observed in preclinical and clinical colorectal cancer models [50]. Our results described herein reveal the biological cellular program(s) and mechanism(s) underlying CD36-mediated anti-HER2 drug refractoriness and poor prognosis in this oncometabolic scenario, which were recently described in HER2 + BC in both preclinical and clinical settings [11, 13].

Several studies have suggested that CD36 plays an important role in the biological activities of CSCs in distinct oncotypes [12, 14, 22, 23, 39]. CSCs exist in MET- and EMT-like states, which rely on different metabolic pathways, highlighting the idea that exploiting the metabolic vulnerabilities of distinct BCSC states is a novel therapeutic approach for targeting this critical tumor cell population [51]. Accordingly, we observed that CD36 expression was strikingly high in HER2 + BC cases characterized by high expression of the basal marker CD44v6 [52], revealing enrichment of CD36 in tumor cells characterized by a mesenchymal/basal-like phenotype. Concurrently, tumors with a predominant epithelial phenotype, *i.e.,* high expression of the MET-CSC marker ALDH1A1 [19], showed markedly lower expression of CD36. These findings were reflected in all tested in vitro cell models, where a more detailed analysis of the CSC subpopulations involved was more technically feasible. Indeed, we found that CD36 was highly expressed in the BCSC population with the CD44^High^/CD24^Low^ phenotype compared with the bulk cell population, while ALDH + cells exhibited lower levels of CD36 expression than did the total differentiated/bulk tumor cell population. Unsurprisingly, EMT-like CSCs also demonstrated a greater capacity to internalize FAs from the extracellular environment. These data strongly indicate the existence of differences in lipid metabolism among the different HER2 + cell populations. Specifically, a high CD36 level leads to an increase in the intracellular FA content, especially that of palmitoyl-CoA. A greater capacity to internalize FAs, coupled with lower activity of the HER2-FASN axis, could lead to increased activity of the catabolic FA oxidation (FAO) pathway; increased FAO activity is associated with aggressive tumor features in BC [53], revealing a potentially new lipid metabolic vulnerability via the specific targeting of carnitine palmitoyl transferase 1 (CPT1A) with etomoxir/ST1326 inhibitors, which are already used clinically [54, 55].

Our findings not only indicate that CD36 is a marker of HER2 + CSCs characterized by a unique EMT-like phenotype but also reveal that upregulation of CD36 may induce an imbalance in the neoplastic stem cell state, promoting a switch toward an EMT-like phenotype. Considering the limited information available on the biological mechanisms underlying the dynamic cellular plasticity between the EMT-like and MET-like states, CD36 could emerge as an active metabolic biomarker for therapeutic resistance, and in turn, its specific inhibition in HER2 + BC cells could promote a MET-like stemness phenotype, consequently increasing sensitivity to anti-HER2 drugs, as demonstrated by our preclinical data. However, currently, there are no molecules safe for in vivo use that can effectively inhibit CD36 protein activity [12], and understanding the functional mechanism that regulates CD36 expression, which has not yet been fully elucidated, could allow the development of a useful therapeutic strategy for indirectly blocking CD36 activity and FA uptake in tumor cells. Therefore, we focused our efforts on investigating the involvement of signaling pathways implicated in stem cell maintenance/survival, such as the Wnt, Notch, Hedgehog, JAK/STAT, TGFβ and NF-kB pathways [26, 43, 44], and in the regulation of CD36 expression. Our data clearly demonstrate that CD36 expression is regulated by Wnt signaling. Accordingly, Wnt/ catenin beta 1 signaling has been implicated in resistance to trastuzumab and lapatinib, and its inhibition is consistent with the effectiveness of anti-HER2 treatment [44]. Outside the CSC setting but consistent with our findings, a functional link between CD36 and the Wnt pathway has been described in cancer cells, but conflicting data have been obtained [56, 57]. Furthermore, the Wnt pathway appears to modulate the CD36 level mainly in therapy-resistant HER2 + BC cell lines characterized by decreased baseline expression of CD36 (HCC1954/EFM192A *vs.* MDAMB361/HCC1569), where CD36 expression is likely restricted to the CSC population, in which it leads to increased MS formation. This observation supports the hypothesis that this regulatory pathway is involved in the control of CD36 expression specifically in the EMT-like stem cell compartment.

We investigated the therapeutic implications of CD36 inhibition in HER2 + BC and found that dual CD36 and HER2 blockade significantly enhanced the therapeutic benefit of HER2 blockade both in vitro and in vivo by targeting HER2 +/CD36 + EMT-like stem-like cells in lapatinib-refractory cell models and by reducing tumor growth in mice implanted with CD36-silenced MDAMB361 cells compared with mice implanted with control cells. Our data biologically explain the preclinical findings obtained by Feng and colleagues, who showed the advantage of concomitant inhibition of CD36 and HER2 over monotherapy in reducing tumor cell proliferation [11], and offer mechanistic support for the results of our previous clinical study describing the outcome of CD36+ HER2 + BC patients receiving neoadjuvant trastuzumab [13]. In particular, the evidence of intratumor enrichment of HER2/CD36/CD44v6-coexpressing stem-like cells in pre-neoadjuvant treatment biopsies from HER2 + BC patients who did not achieve pCR compared with those who achieved pCR clearly underscores the crucial role played by CD36 in HER2 inhibitor resistance in the HER2 + BC oncotype and emphasizes how CD36 inhibition influences parameters such as invasiveness, relapse, metastatic potential and stemness [14].

Additionally, while our findings provide significant evidence of CD36 role in FA uptake and its enrichment in HER2 + EMT-like CSCs, certain limitations must be acknowledged.

The main limitation of this study is that we performed molecular analysis on only a limited number of BC patients (INT-MI series) who received neoadjuvant trastuzumab-based therapy and for whom transcriptomic data from basal tumor biopsies were available. However, the data from our study cohort were partially validated in a neoadjuvant window-of-opportunity study, enabling us to evaluate the biological effects of trastuzumab independently without the potential confounding effects of the associated chemotherapy backbone [31]. Based on the rapid response to HER2 blockade by trastuzumab in modulating CD36 in HER2 + BC observed in this trial —regardless of their tumor sensitivity to treatment—further research is warranted to clarify the functional role of the rapid increase in CD36 expression compared with the changes observed with long-term neoadjuvant anti-HER2 treatment, which we demonstrated to be associated with CD36-driven FA uptake and the emergence of a mesenchymal-like CSC phenotype promoting resistance. Further studies will focus on gaining a more detailed understanding of why the reprogramming of FA uptake induces plasticity in HER2 + mammary tumor cells, leading to the intratumor enrichment of EMT-like CD36+ /HER2 + cancer stem cells (CSCs). Additionally, investigations will explore whether EMT-like HER2 + CSCs exhibit metabolic dependency on CD36-mediated FA uptake. Nevertheless, our present data demonstrate that targeting CD36 in HER2 + BC may favor the transition of CSCs to a trastuzumab-sensitive state, representing an ideal combinatorial approach to boost the anti-CSC activity of trastuzumab and increase treatment efficacy in HER2 + BC patients.

## Conclusions

This study revealed the critical role of CD36-mediated FA uptake in HER2 + therapy-resistant BC and provided evidence of persistent enrichment of CD36 in HER2 + EMT-like CSCs that are refractory to anti-HER2 therapy due to Wnt pathway activation. Indeed, CD36 targeting, favoring the transition of CSCs in a trastuzumab-sensitive state, could represent an ideal combinatorial approach to boost the anti-CSC activity of trastuzumab and thus increase treatment efficacy in HER2 + BC patients.

## Supplementary Information

Supplementary Material 1

Supplementary Material 2

## Data Availability

All the data related to this study are available within the article and its Supplementary Information files. Gene expression data generated in this study are available from the Gene Expression Omnibus (GEO) under accession number GSE245132.

## Abbreviations

7-AAD: 7-Amino-actinomycin D
ACTB: Beta-actin
ALDH: Aldehyde dehydrogenase
ALDH +: Aldehyde dehydrogenase-positive
ALDH-: Aldehyde dehydrogenase-negative
ALDH1A1 +: Aldehyde dehydrogenase family 1 member A1-positive
APC: Allophycocyanin conjugate
ATCC: American Type Culture Collection
BAAA: Bodipy-aminoacetaldehyde
BC: Breast cancer
BCSCs: Breast cancer stem cells
BD: Becton Dickinson
CPT1A: Carnitine palmitoyl transferase 1A
CSCs: Cancer stem cells
CTNNB1: Catenin beta 1
DAB: 3-3’-Diaminobenzidine
DAPI: 4',6-Diamidino-2-phenylindole
DEAB: Diethylaminobenzaldehyde
DMEM: Dulbecco’s modified Eagle’s medium
DMSO: Dimethyl sulfoxide
EMT: Epithelial-mesenchymal transition
FA: Fatty acid
FACS: Fluorescence-activated cell sorting
FASN: Fatty acid synthase
FAO: FA oxidation
FBS: Fetal bovine serum
FFPE: Formalin-fixed paraffin-embedded
FITC: Fluorescein Isothiocyanate conjugate
GEP: Gene expression profile
GFP: Green fluorescent protein
GOBP: Gene ontology biological process
GSEA: Gene set enrichment analysis
GSVA: Gene set variation analysis
HER2 +: HER2-positive
HRP: Polymeric horseradish peroxidase
IHC: Immunohistochemistry
INT: Fondazione IRCCS Istituto Nazionale dei Tumori di Milano, Milan, Italy
m.f.p.: Mammary fat pad
MET: Mesenchymal-epithelial transition
MFE: Mammosphere forming efficiency
MFI: Mean fluorescence intensity
OD: Optical density
PBS: Phosphate buffer solution
pCR: Pathological complete response
PFL: Positive feedback loop
PORCN: Porcupine
PE/Cy7: Phycoerythrin/Cy7-conjugated
qRT-PCR: Quantitative real-time polymerase chain reaction
rMFI: Relative median fluorescence intensity
RPMI1640: Roswell Park Memorial Institute medium
SCID: Severe combined immunodeficient mice
SD: Standard deviation
SEM: Standard error of the mean
shRNA: Short hairpin RNA
shSCR: Scramble shRNA
siRNA: Short interfering RNA
SRB: Sulforhodamine B
SSO: Sulfo N-succinimidoyl Oleate
SST-RMA: Signal space transformation – robust multiarray average
TBS: Tris-buffered saline
TKI: Tyrosine kinase inhibitor
TSA: Tyramide signal amplification
vs.: Versus
