# Correction: CD36 enrichment in HER2-positive mesenchymal stem cells drives therapy refractoriness in breast cancer

**DOI:** 10.1186/s13046-025-03426-3

**Published:** 2025-05-30

**Authors:** Lorenzo Castagnoli, Alma Franceschini, Valeria Cancila, Matteo Dugo, Martina Bigliardi, Claudia Chiodoni, Paolo Toneguzzo, Viola Regondi, Paola A. Corsetto, Filippo Pietrantonio, Serena Mazzucchelli, Fabio Corsi, Antonino Belfiore, Andrea Vingiani, Giancarlo Pruneri, Francesca Ligorio, Mario P. Colombo, Elda Tagliabue, Claudio Tripodo, Claudio Vernieri, Tiziana Triulzi, Serenella M. Pupa

**Affiliations:** 1 Microenvironment and Biomarkers of Solid Tumors Unit, Department of Experimental Oncology, Amadeolab Fondazione IRCCS Istituto Nazionale Dei Tumori Di Milano, Milan, Italy; 2 Tumor Immunology Unit, Department PROMISE, Universita’ Di Palermo, Palermo, Italy; 3 Breast Cancer Unit Clinical Translational and Immunotherapy Research, Department of Medical Oncology, IRCCS Ospedale San Rafaele, Milan, Italy; 4 Molecular Immunology Unit, Department of Experimental Oncology, Amadeolab Fondazione IRCCS Istituto Nazionale Dei Tumori Di Milano, Milan, Italy; 5 Department of Pharmacologicaland, Biomolecular Sciences “Rodolfo Paoletti”,, Università Di Milano, Milan, Italy; 6 Medical Oncology Department, Fondazione IRCCS Istituto Nazionale Dei Tumori Di Milano, Milan, Italy; 7 Department of Biomedical and Clinical Sciences, Università Di Milano, Milan, Italy; 8 Surgery Department, Istituti Clinici Scientifci Maugeri IRCCS, Pavia, Italy; 9 Department of Diagnostic Pathology and Laboratory Medicine, Fondazione IRCCS Istituto Nazionale Dei Tumori Di Milano, Milan, Italy; 10 Department of Oncology and Hemato-Oncology, Universita’ Di Milano, Milan, Italy; 11 IFOM ETS, The AIRC Institute of Molecular Oncology, Milan, Italy

**Correction: J Exp Clin Cancer Res 44, 19 (2025)**

**https://doi.org/10.1186/s13046-025-03276-z**

Following the publication of the original article [1], the authors identified an errors in the author names of Antonino Belfiore and Andrea Vingiani. The correct names are presented below:

The incorrect author name is: Antonio Belfiore

The correct author name is: Antonino Belfiore

The incorrect author name is: Antonio Vingiani

The correct author name is: Andrea Vingiani

The author group has been updated above and the original article [1] has been corrected.
