# Supplementary material for: CD36 enrichment in HER2-positive mesenchymal stem cells drives therapy refractoriness in breast cancer
Source: J Exp Clin Cancer Res. 2025 Jan 20;44:19. doi: 10.1186/s13046-025-03276-z (PMC11744895; doi:10.1186/s13046-025-03276-z)

Supplementary Fig. S1

A

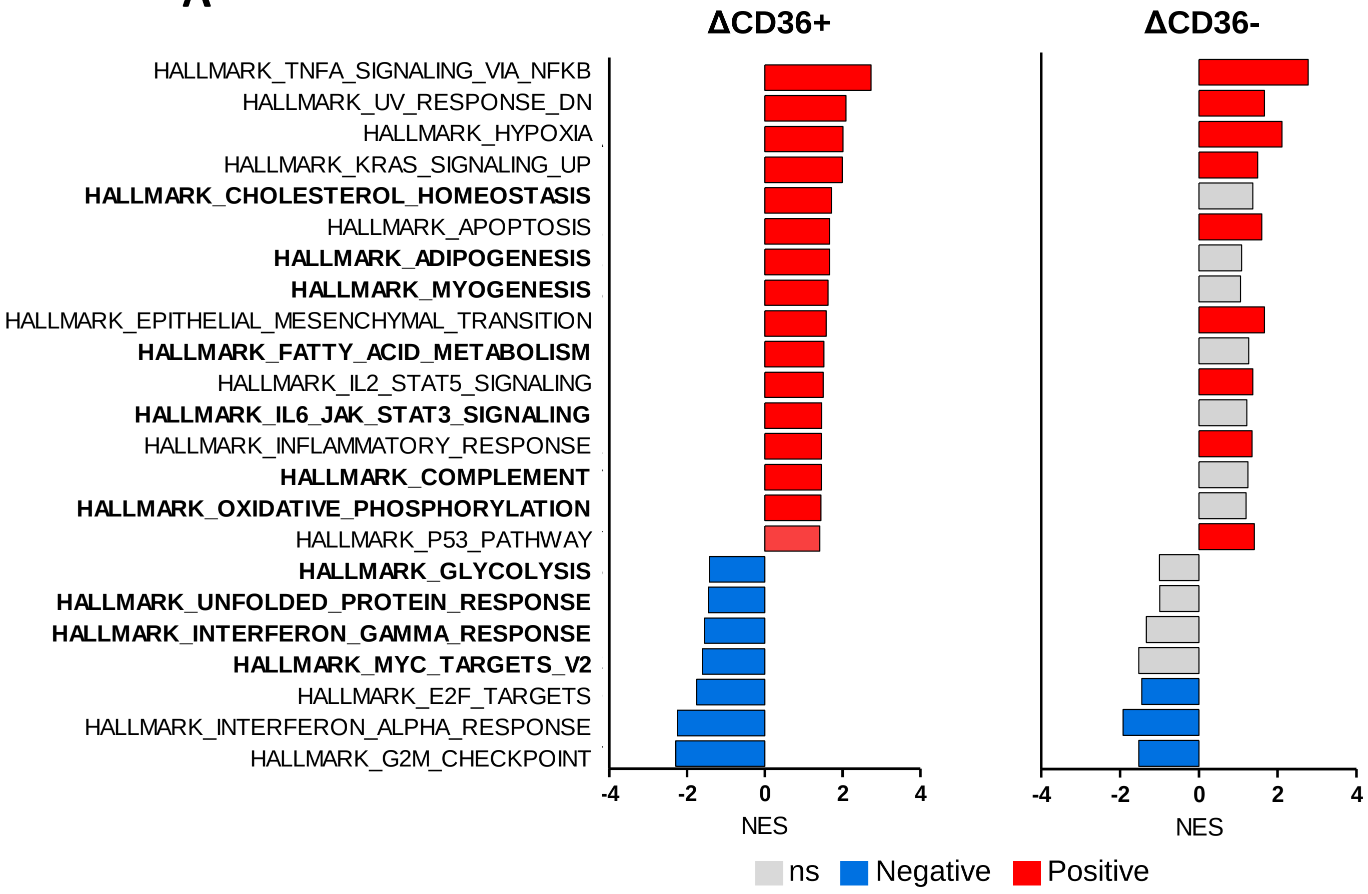

B

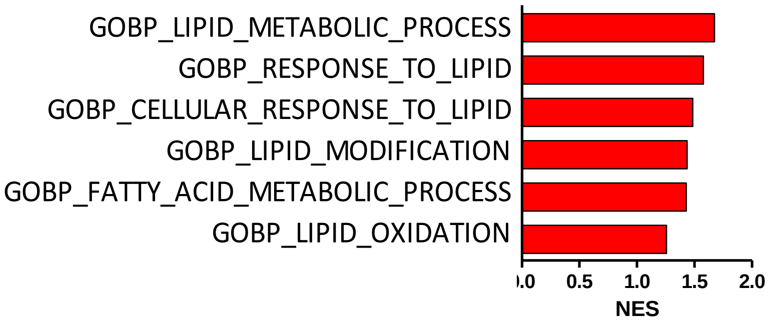

Supplementary Fig. S2

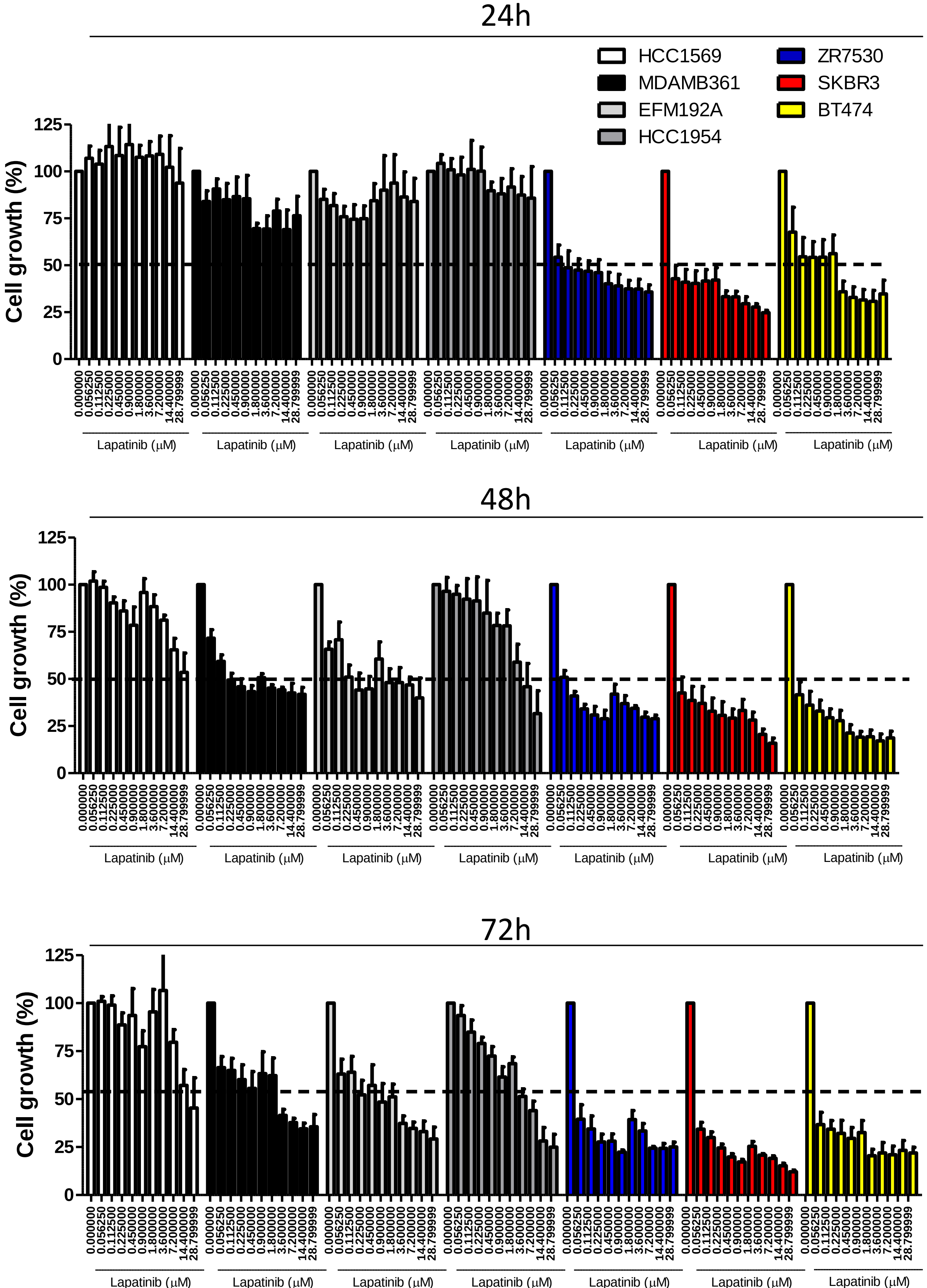

Supplementary Fig. S3

A

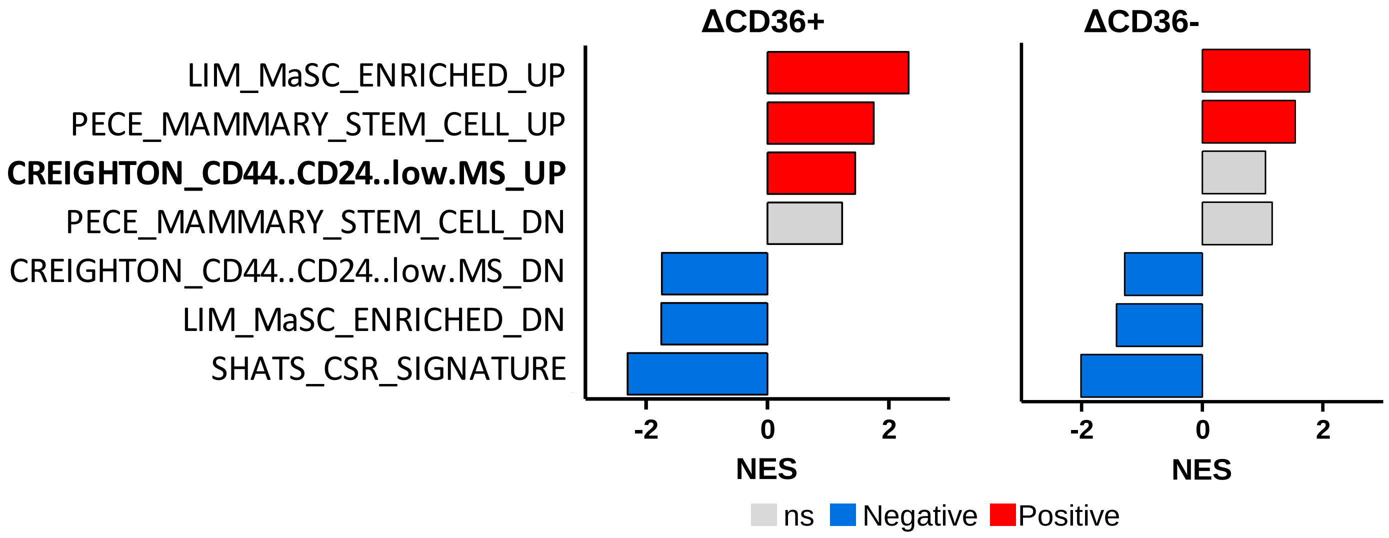

B

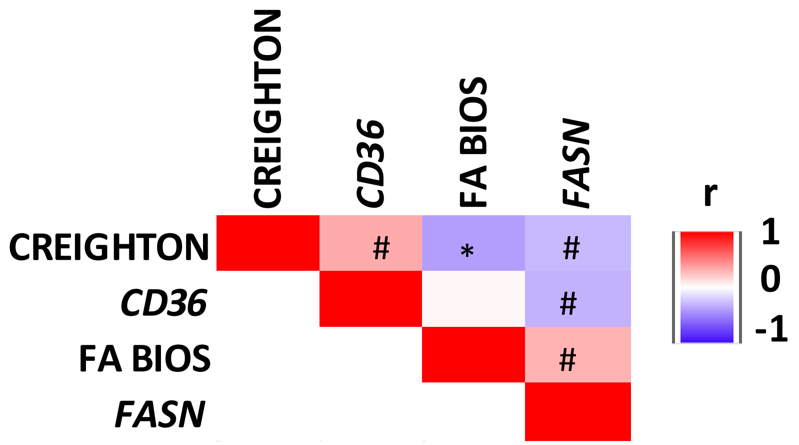

Supplementary Fig. S4

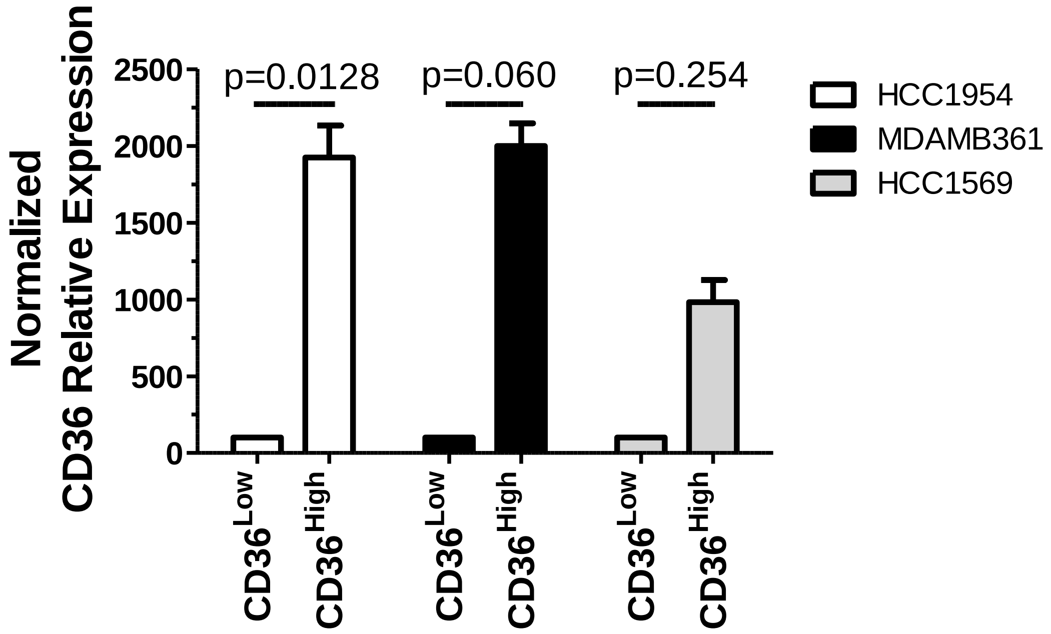

Supplementary Fig. S5

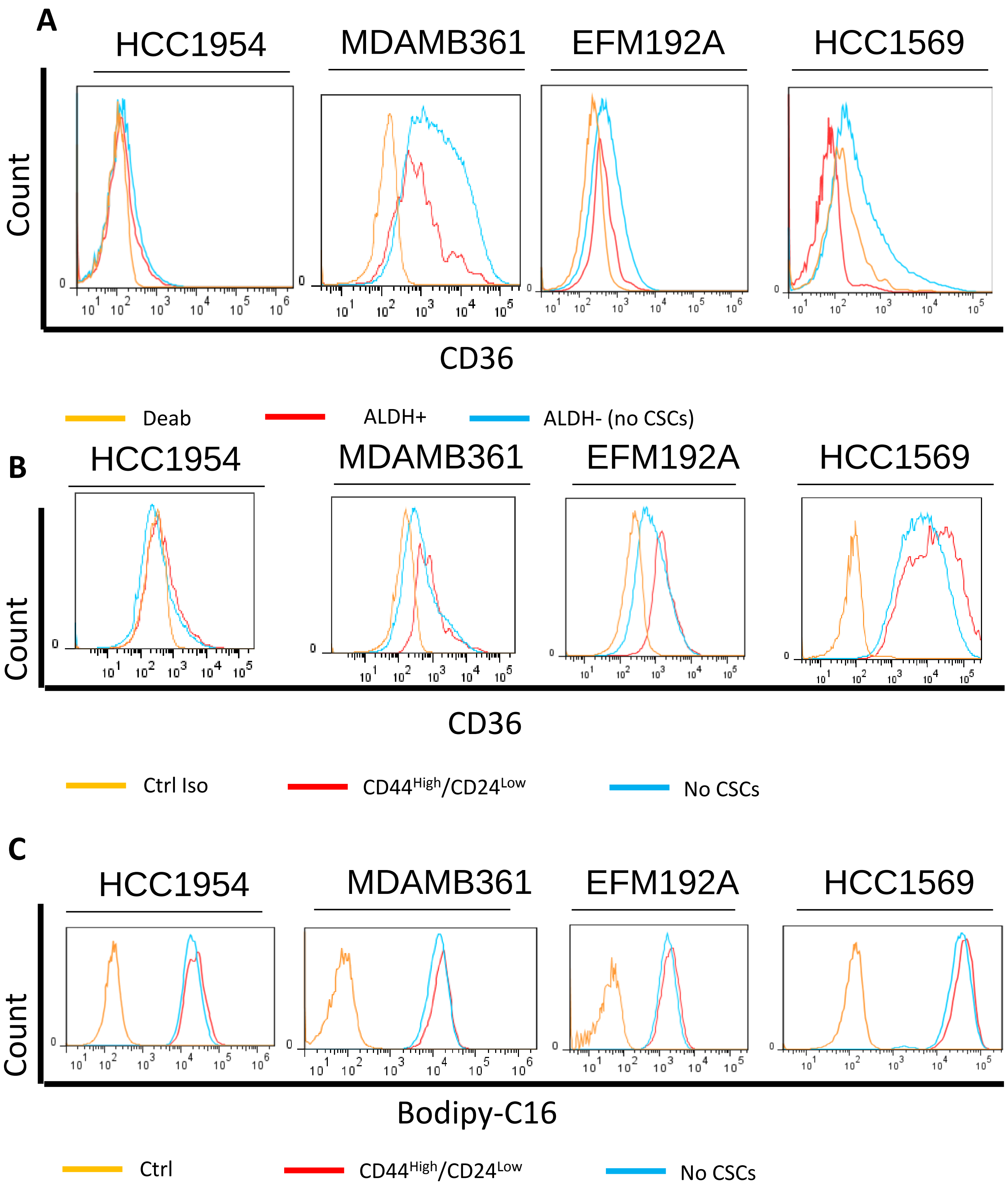

Supplementary Fig. S6

A

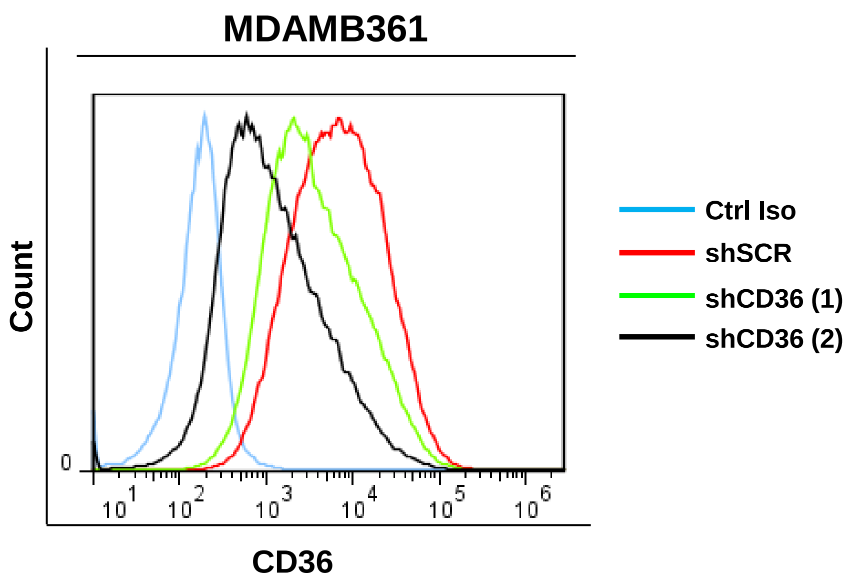

B

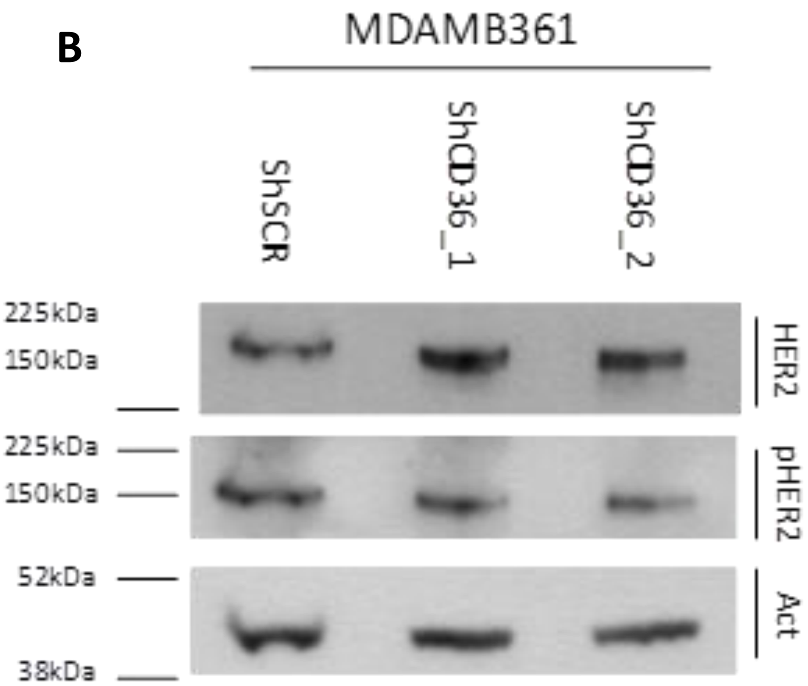

Supplementary Fig. S7

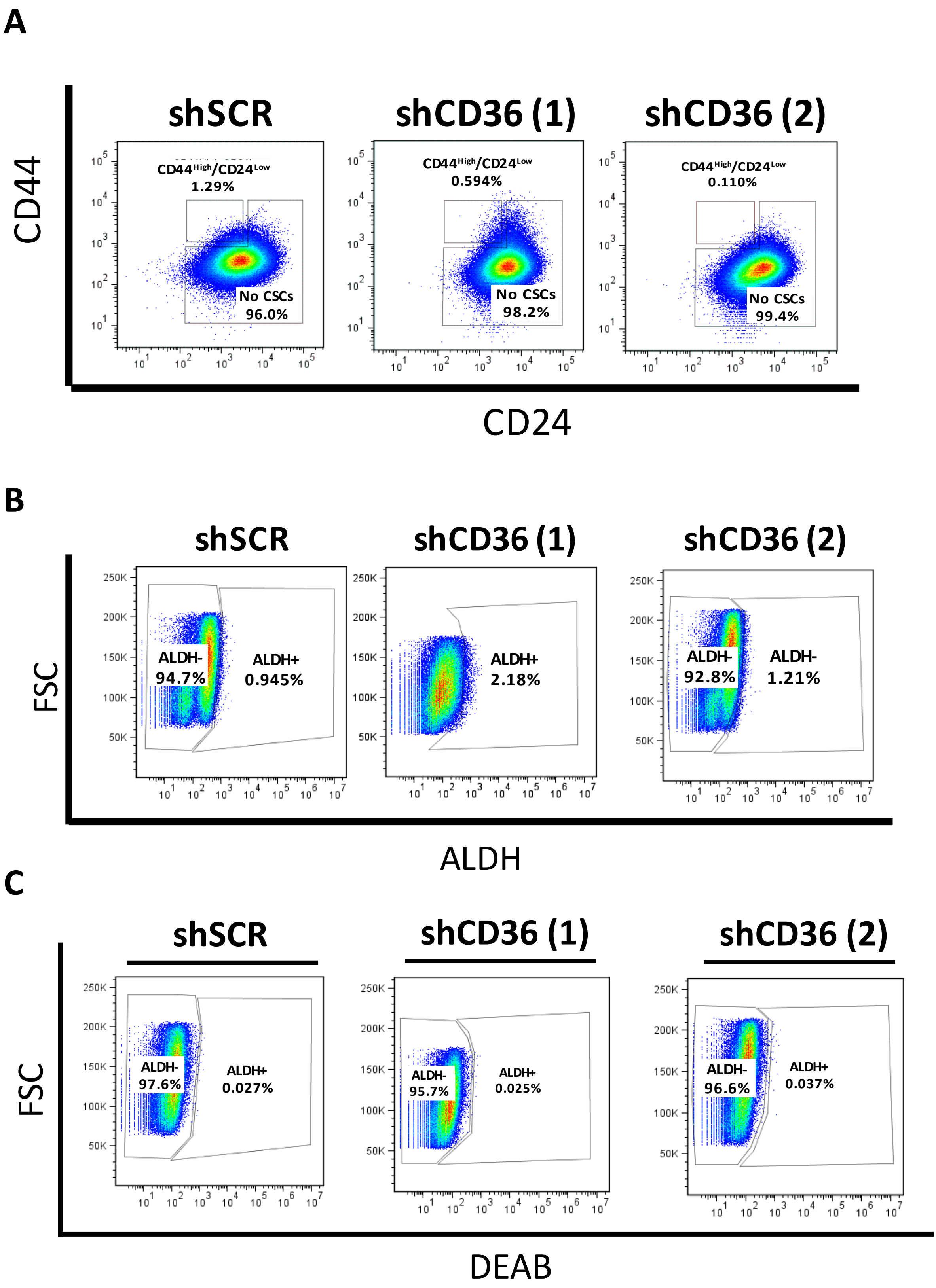

Supplementary Fig. S8

MDAMB361 – 3D

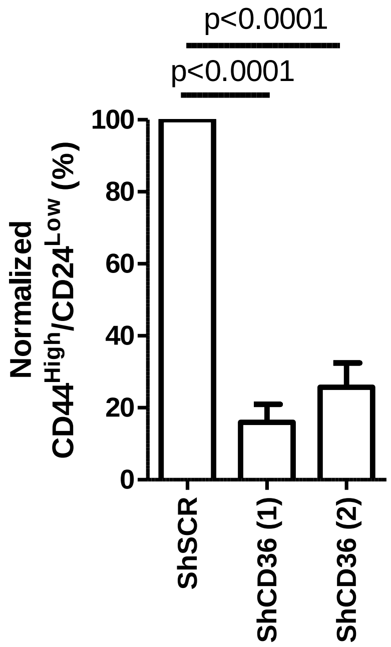

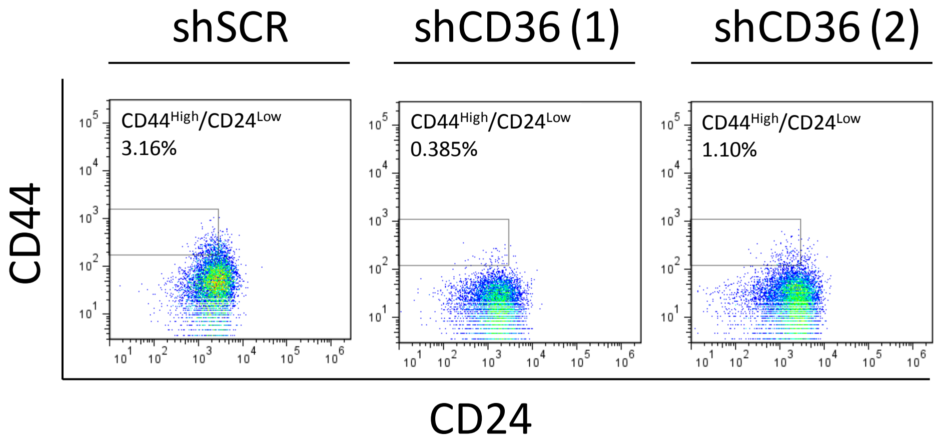

Supplementary Fig. S9

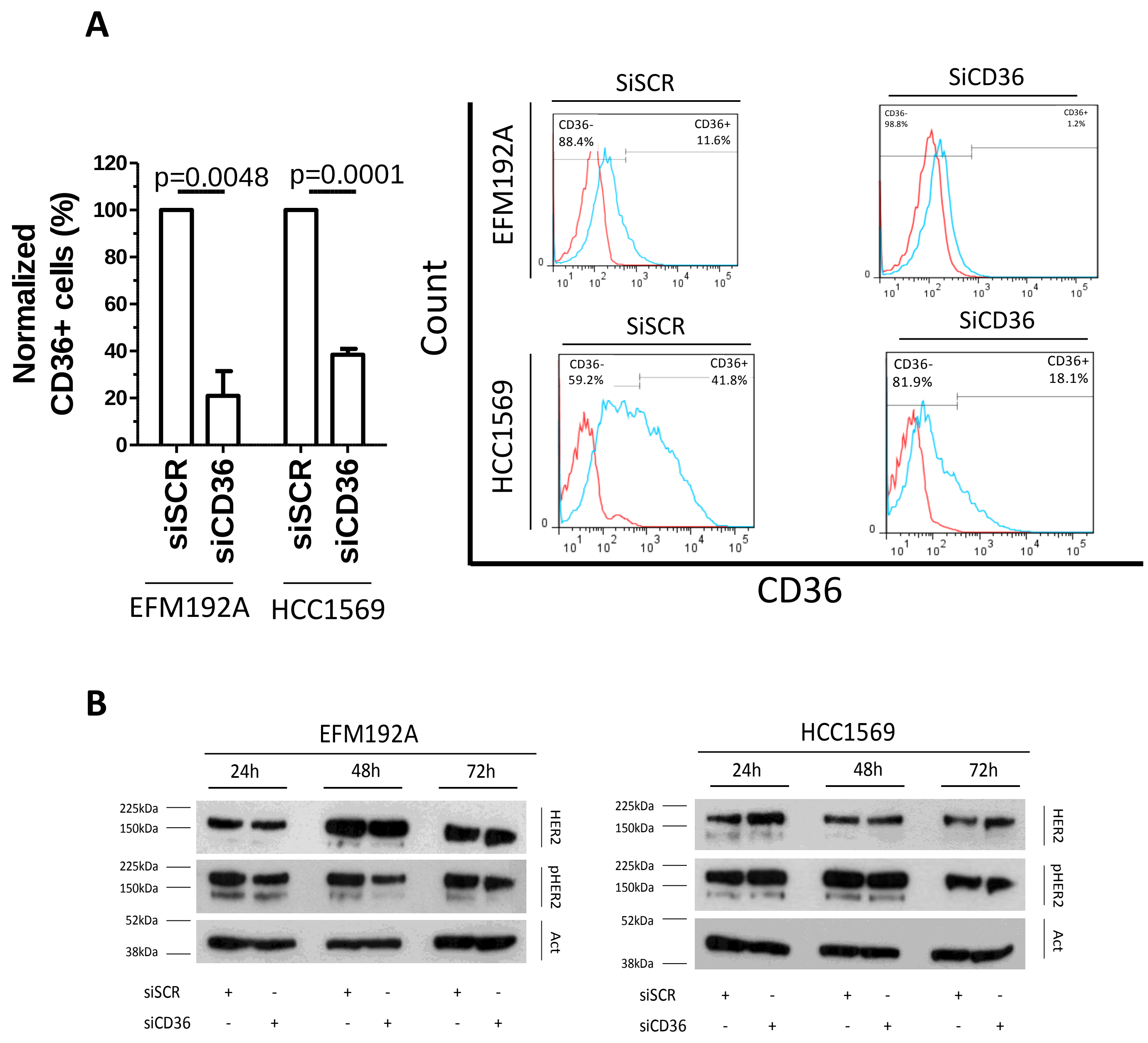

Supplementary Fig. S10

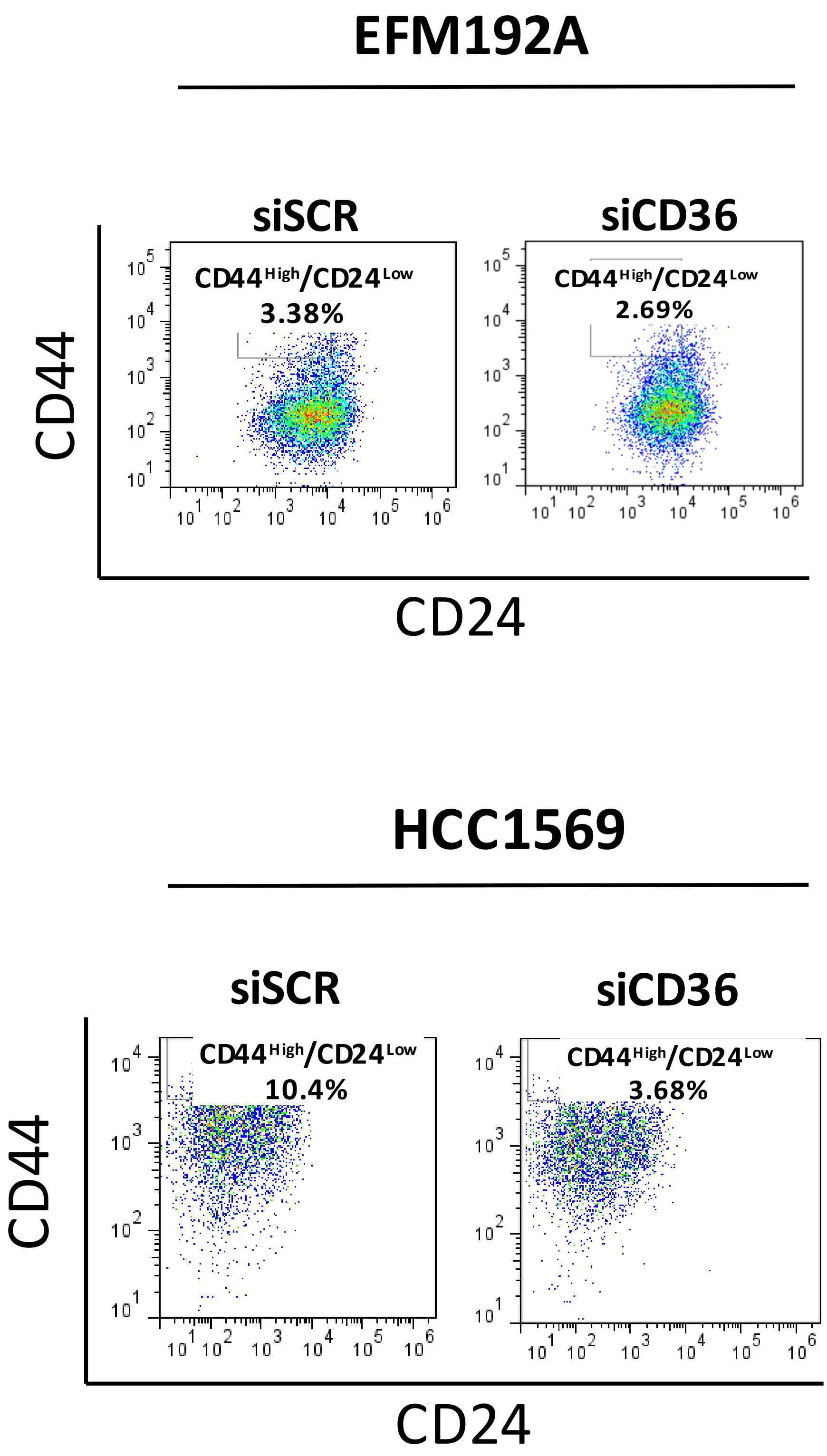

Supplementary Fig. S11

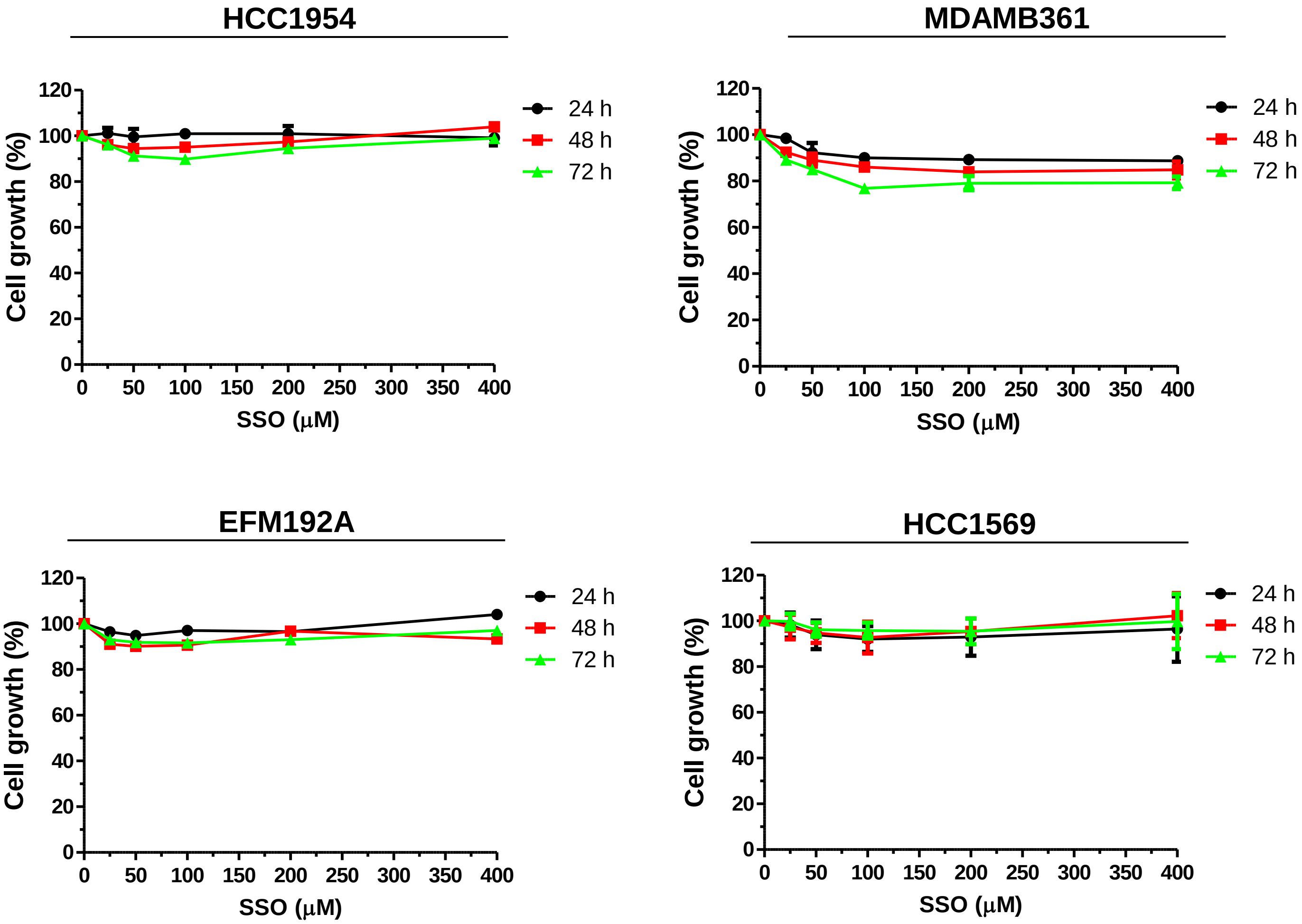

Supplementary Fig. S12

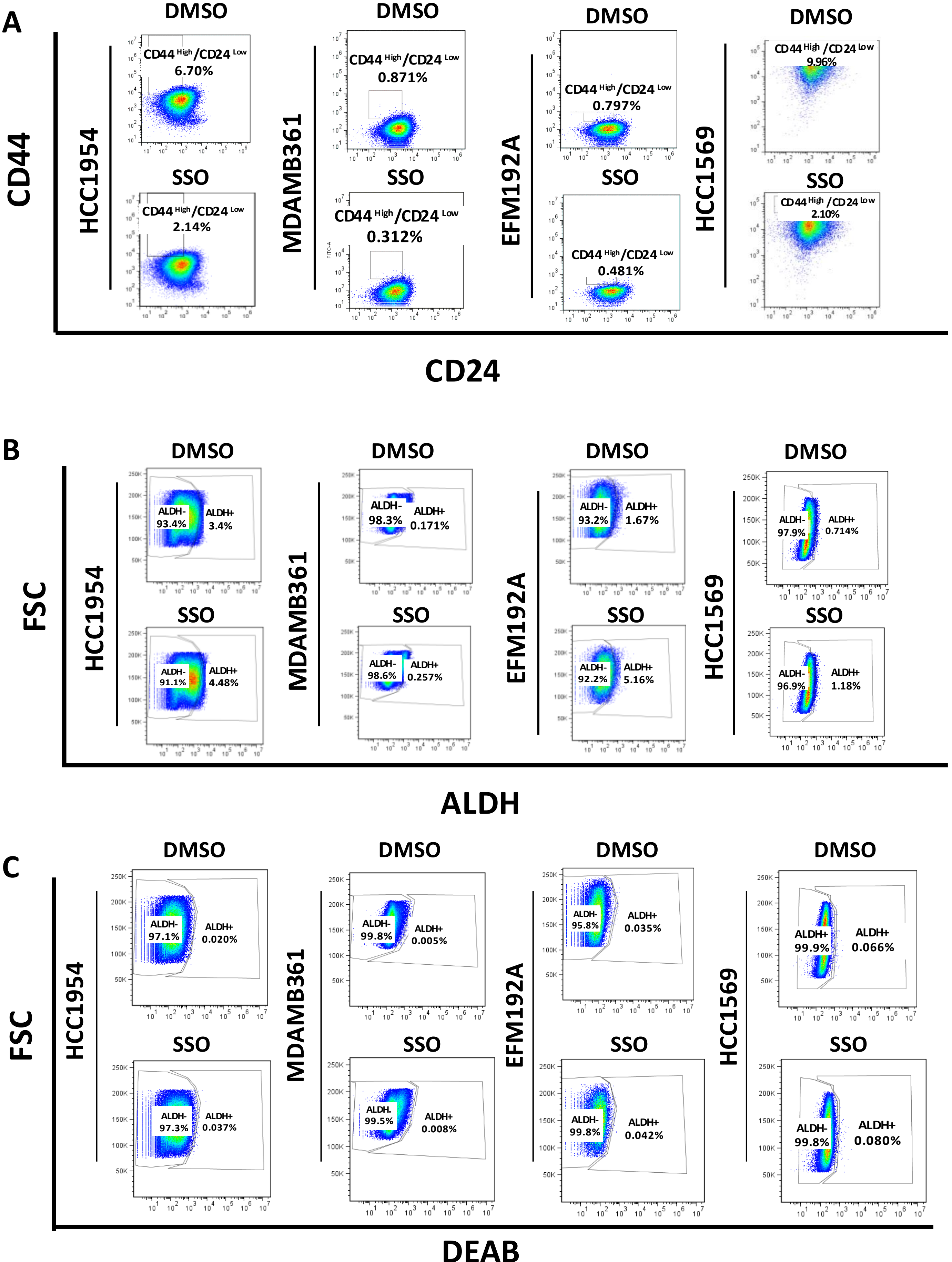

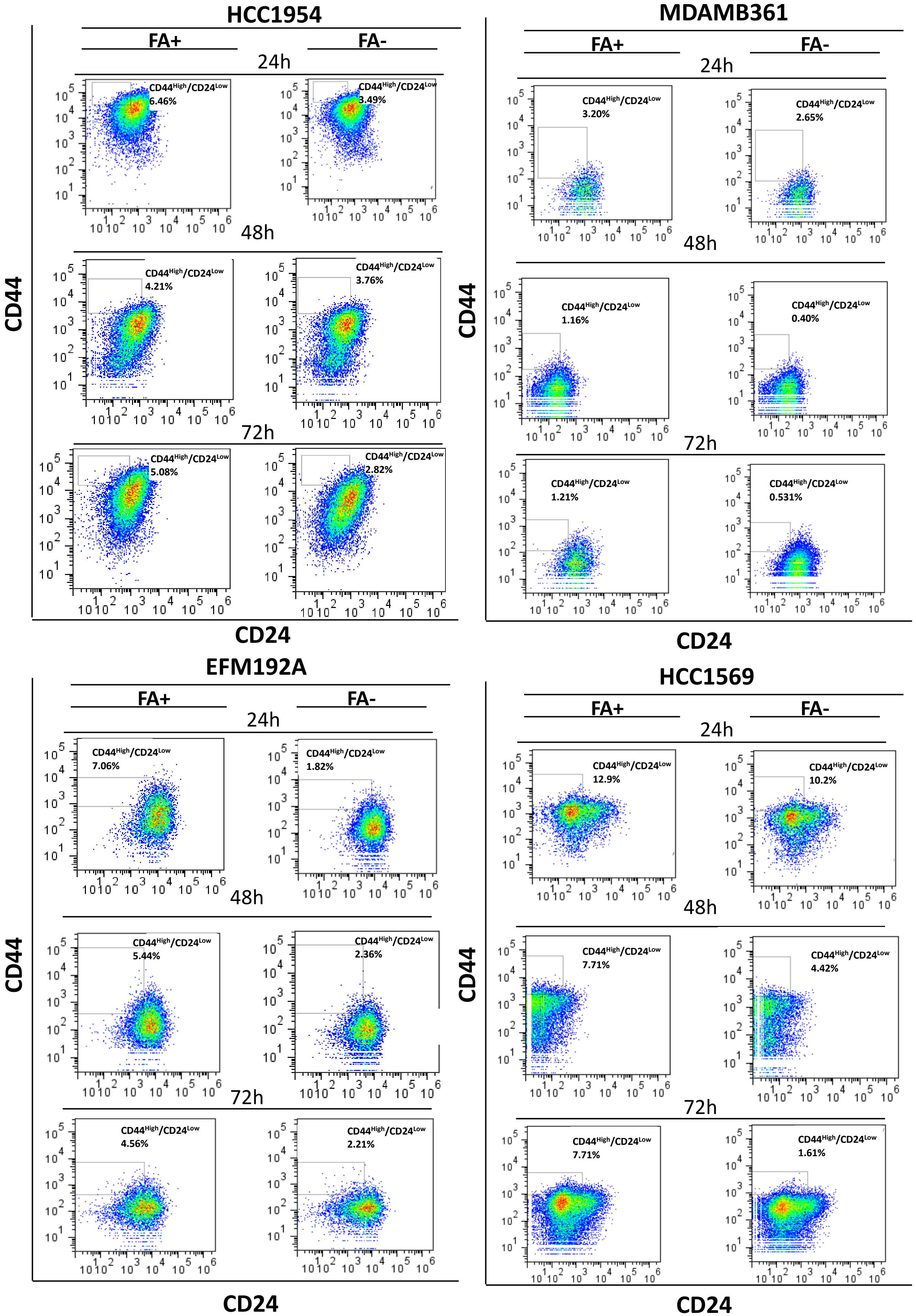

Supplementary Fig. S13

Supplementary Fig. S14

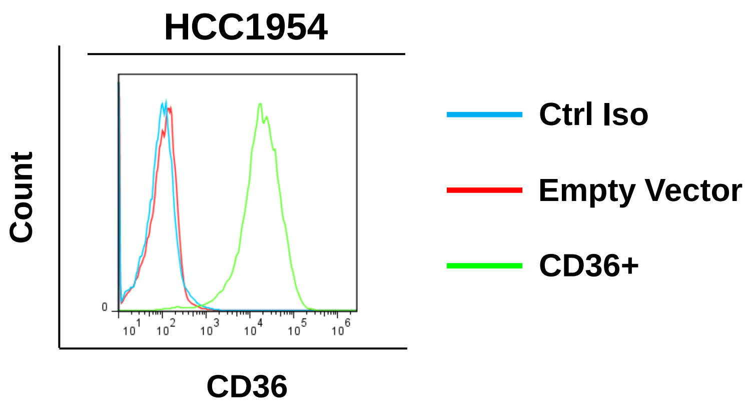

Supplementary Fig. S15

HCC1954

A

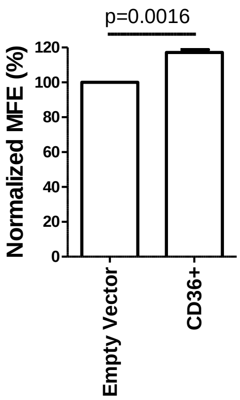

B

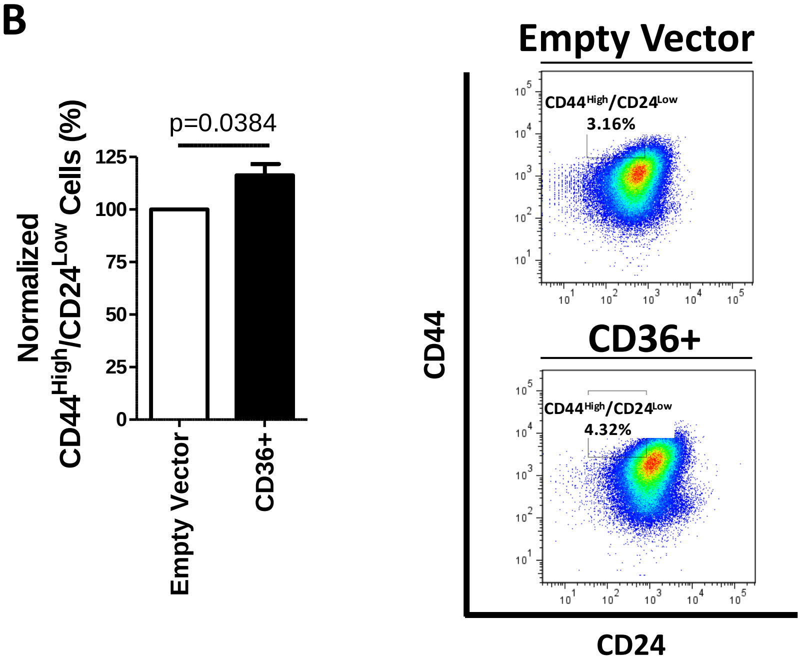

Supplementary Fig. S16

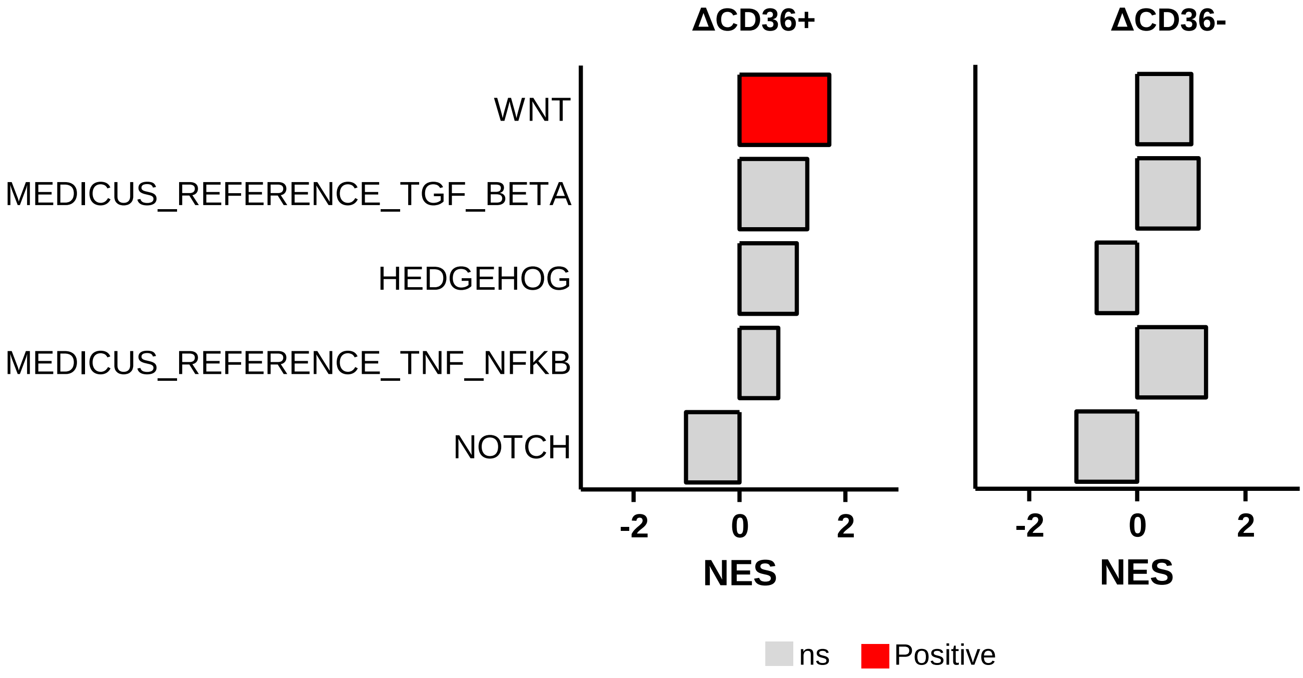

Supplementary Fig. S17

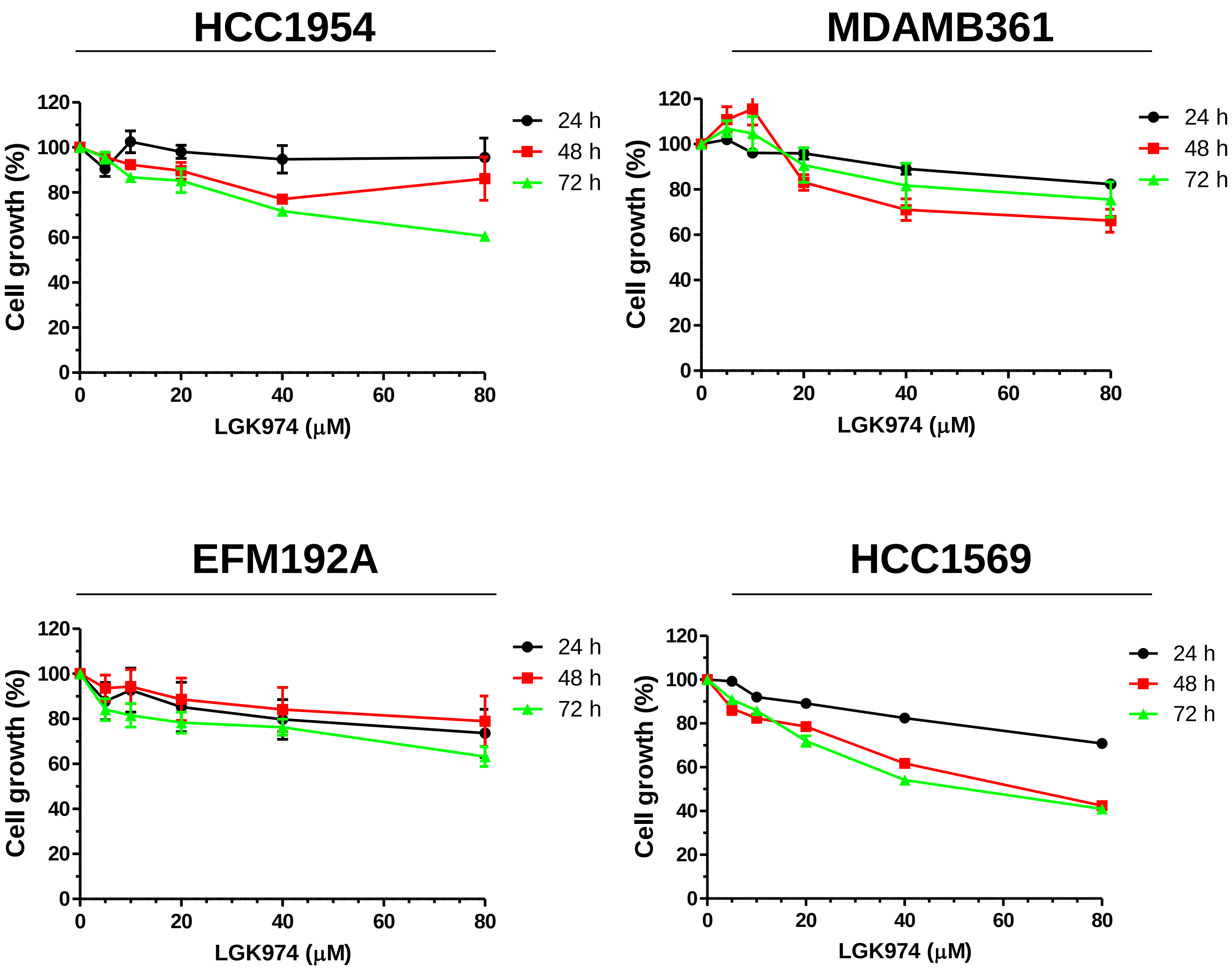

Supplementary Fig. S18

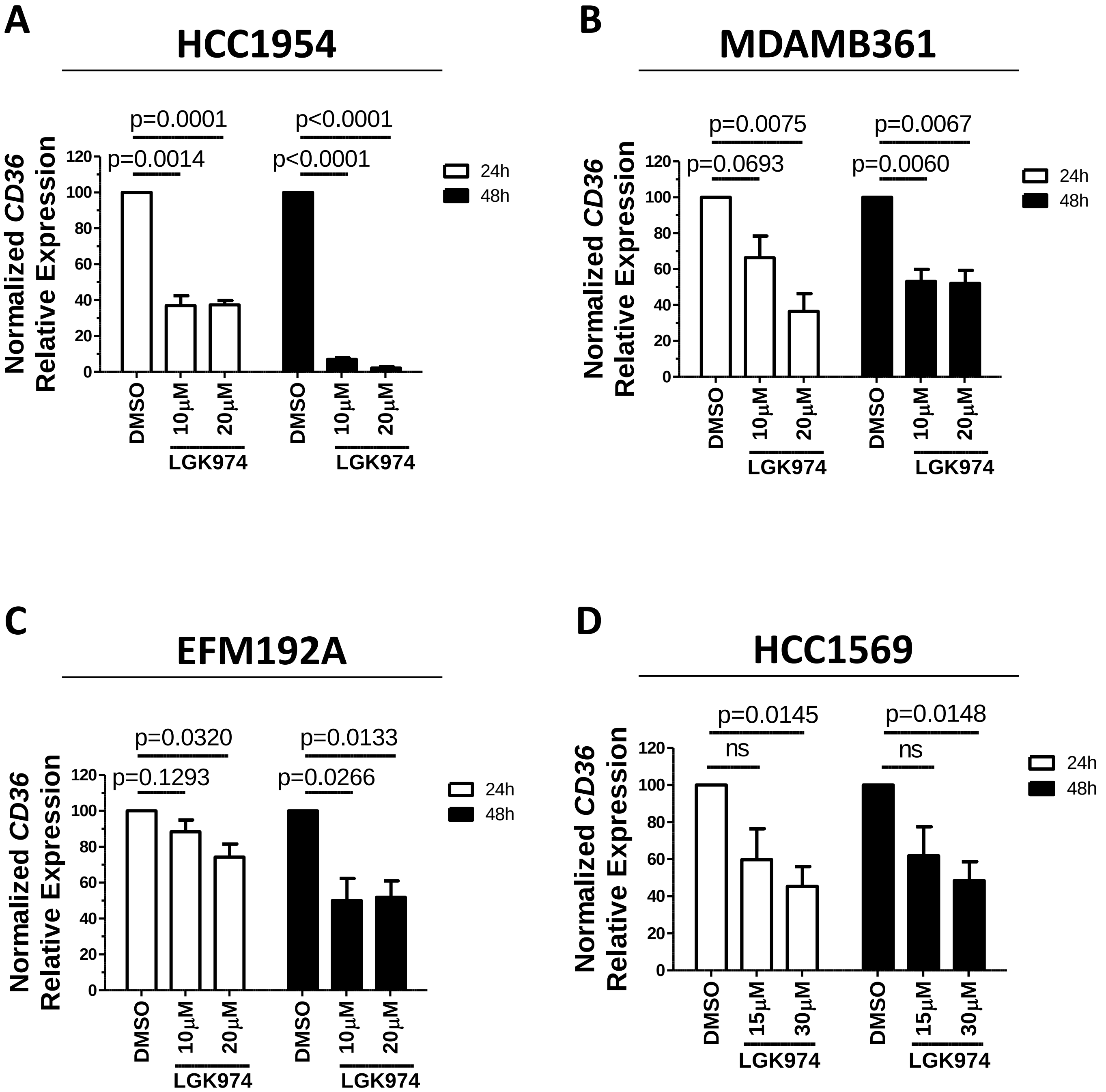

Supplementary Fig. S19

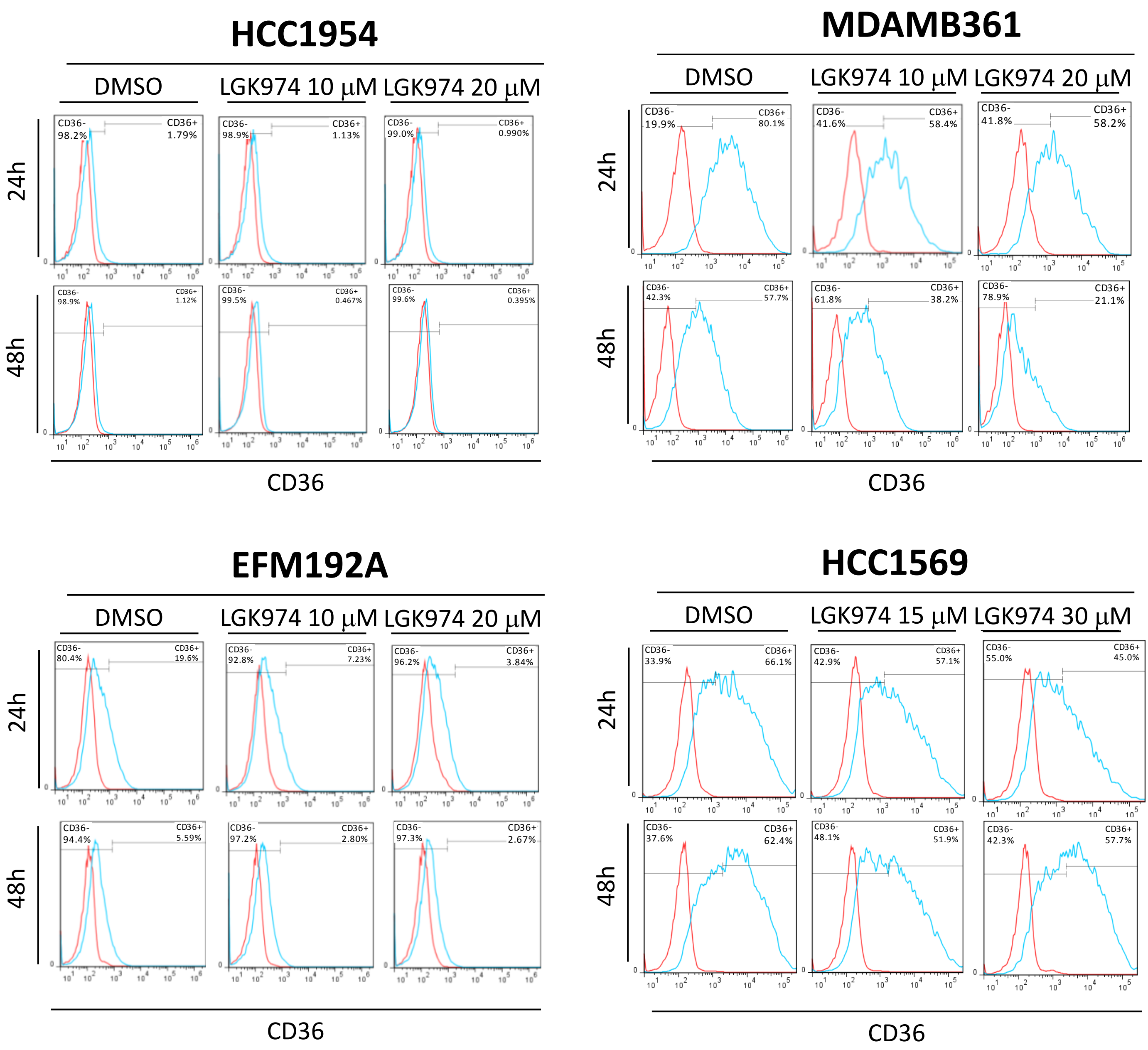

Supplementary Fig. S20

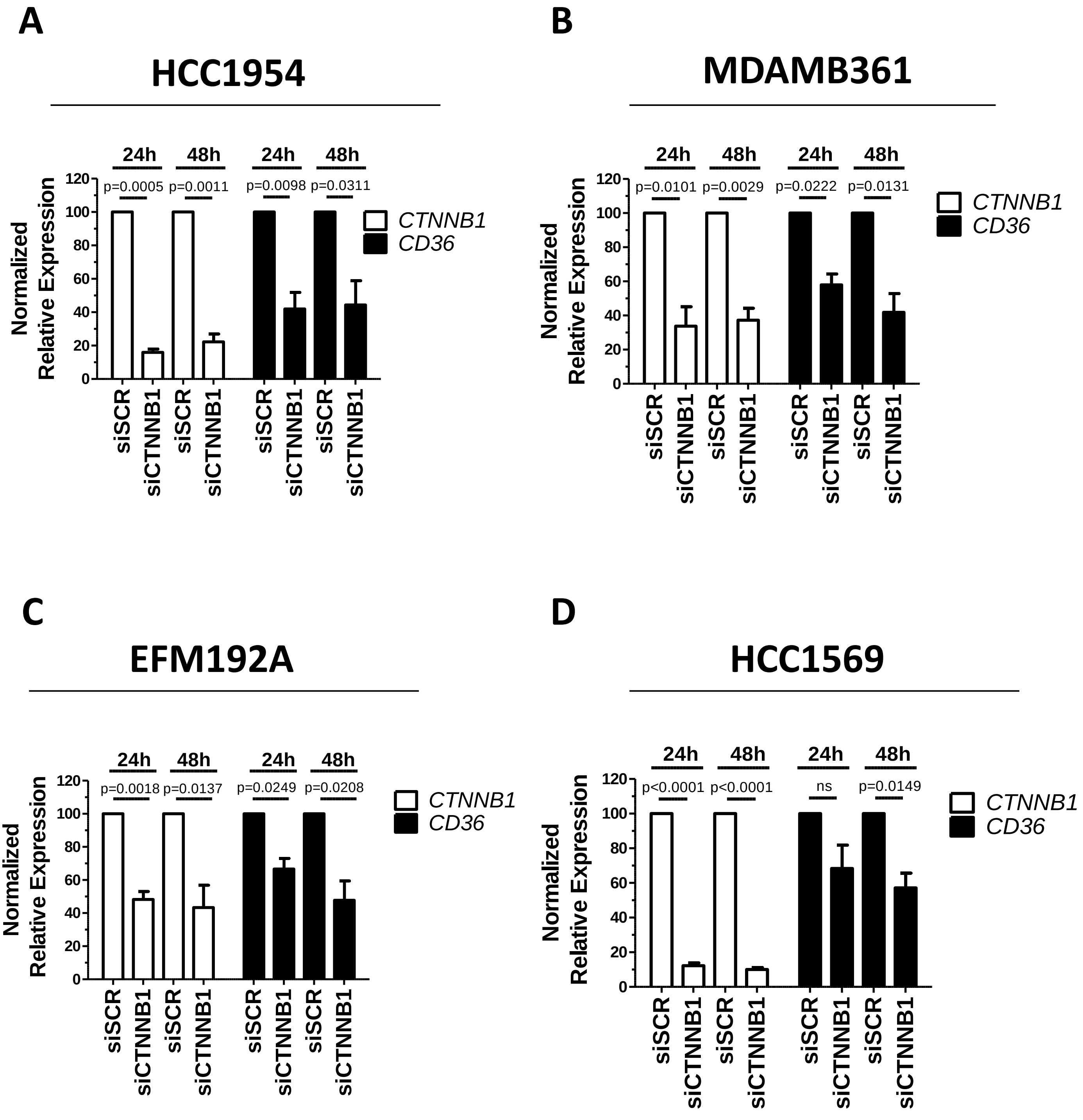

Supplementary Fig. S21

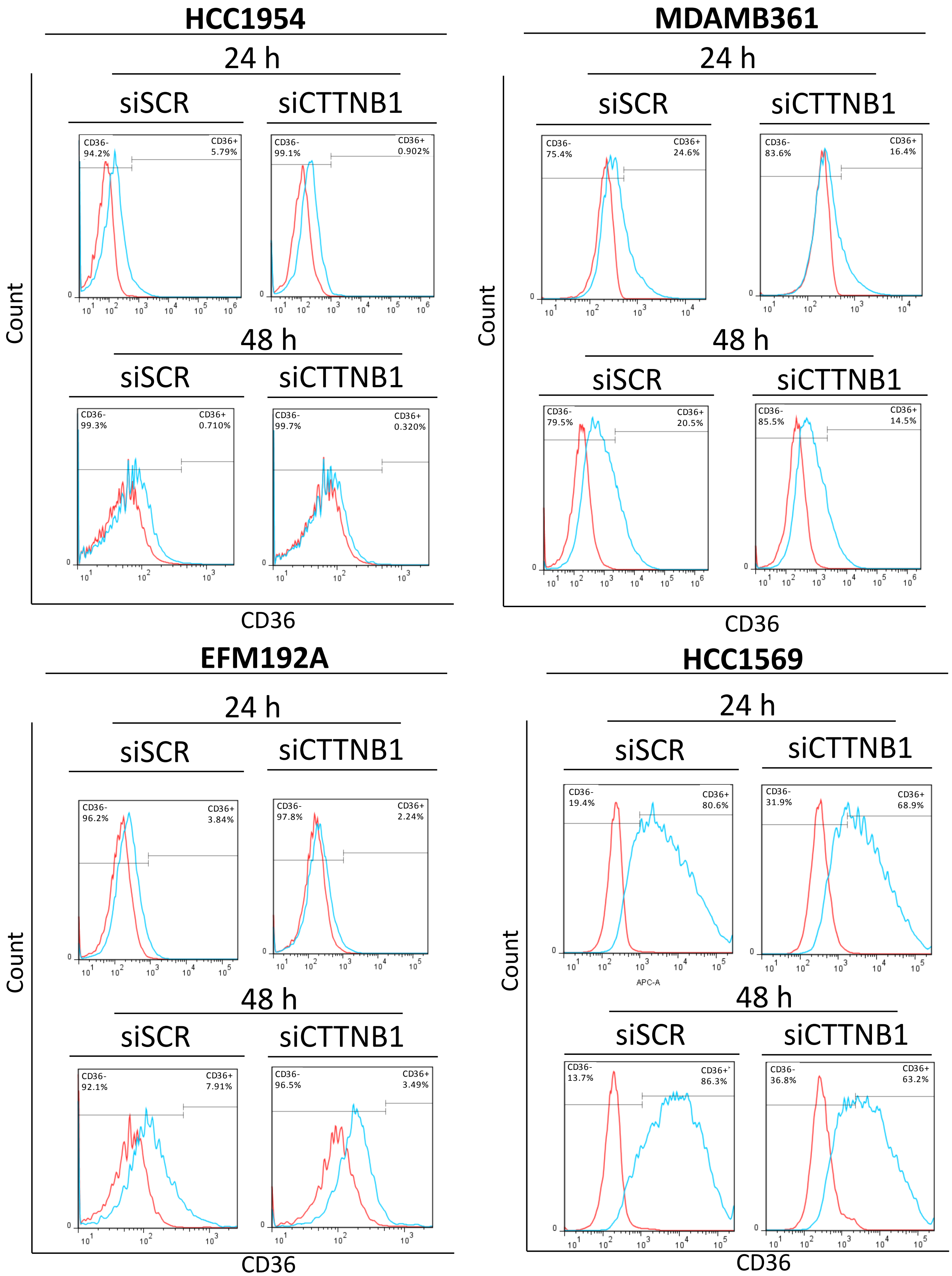

Supplementary Fig. S22

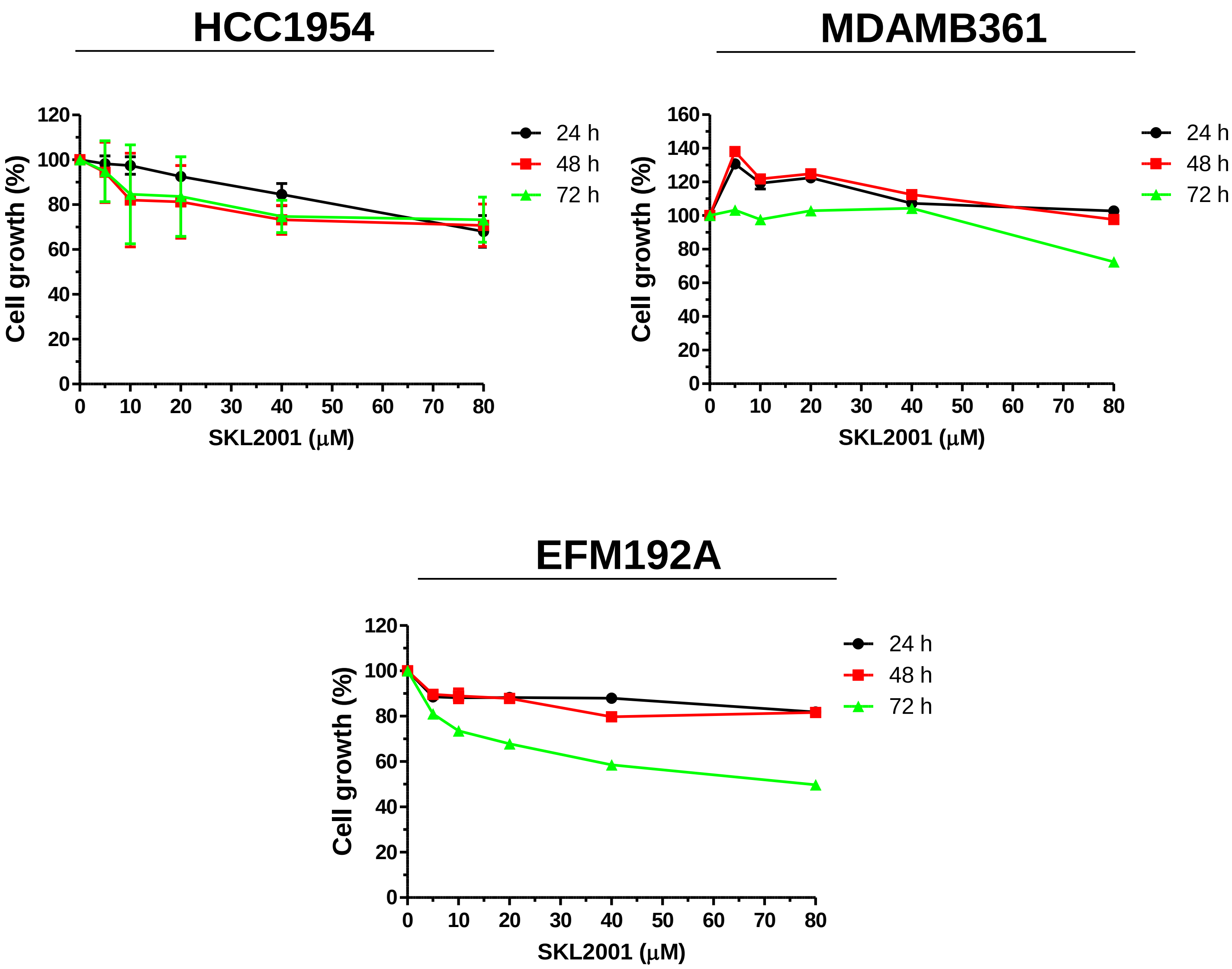

Supplementary Fig. S23

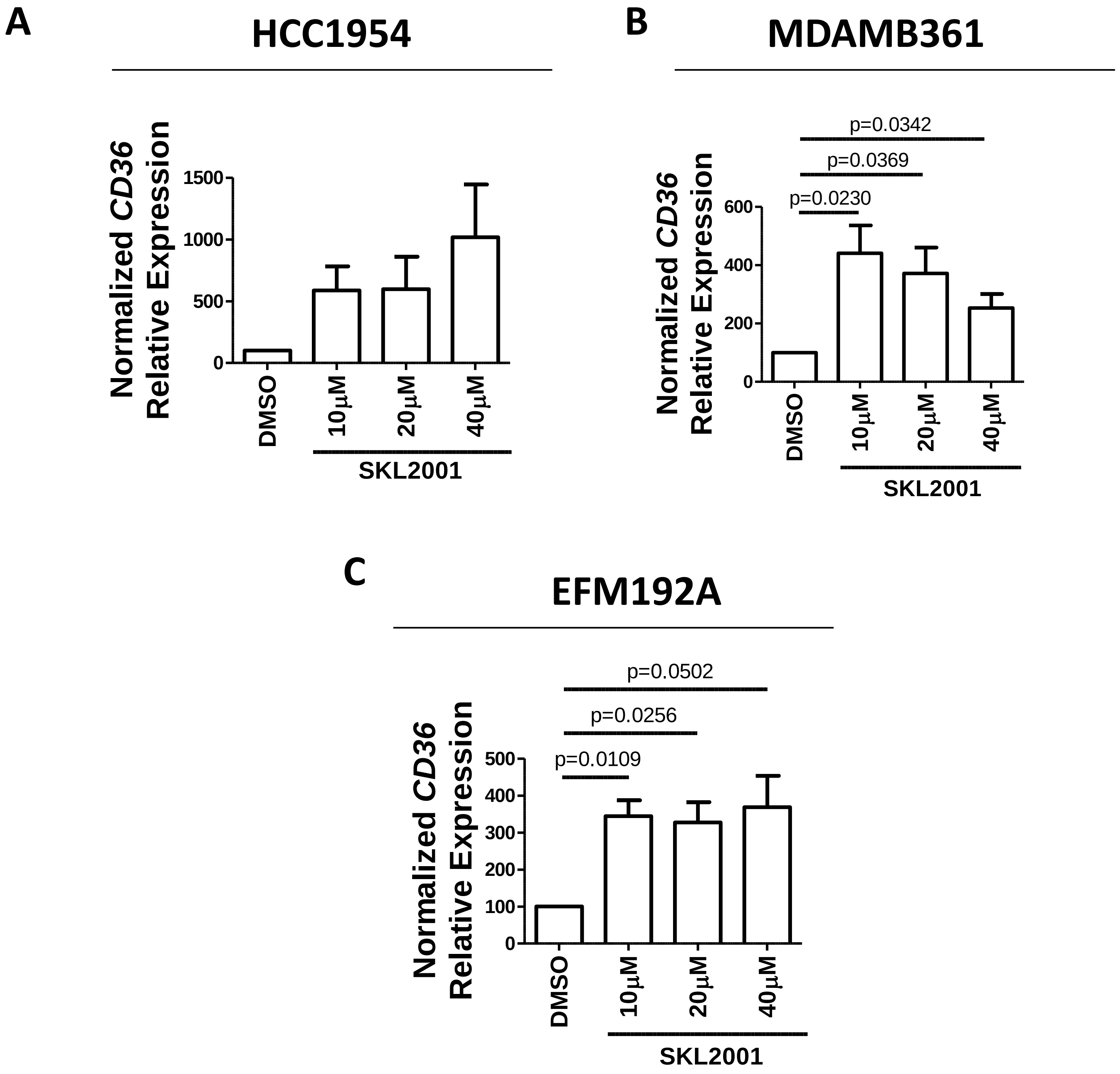

Supplementary Fig. S24

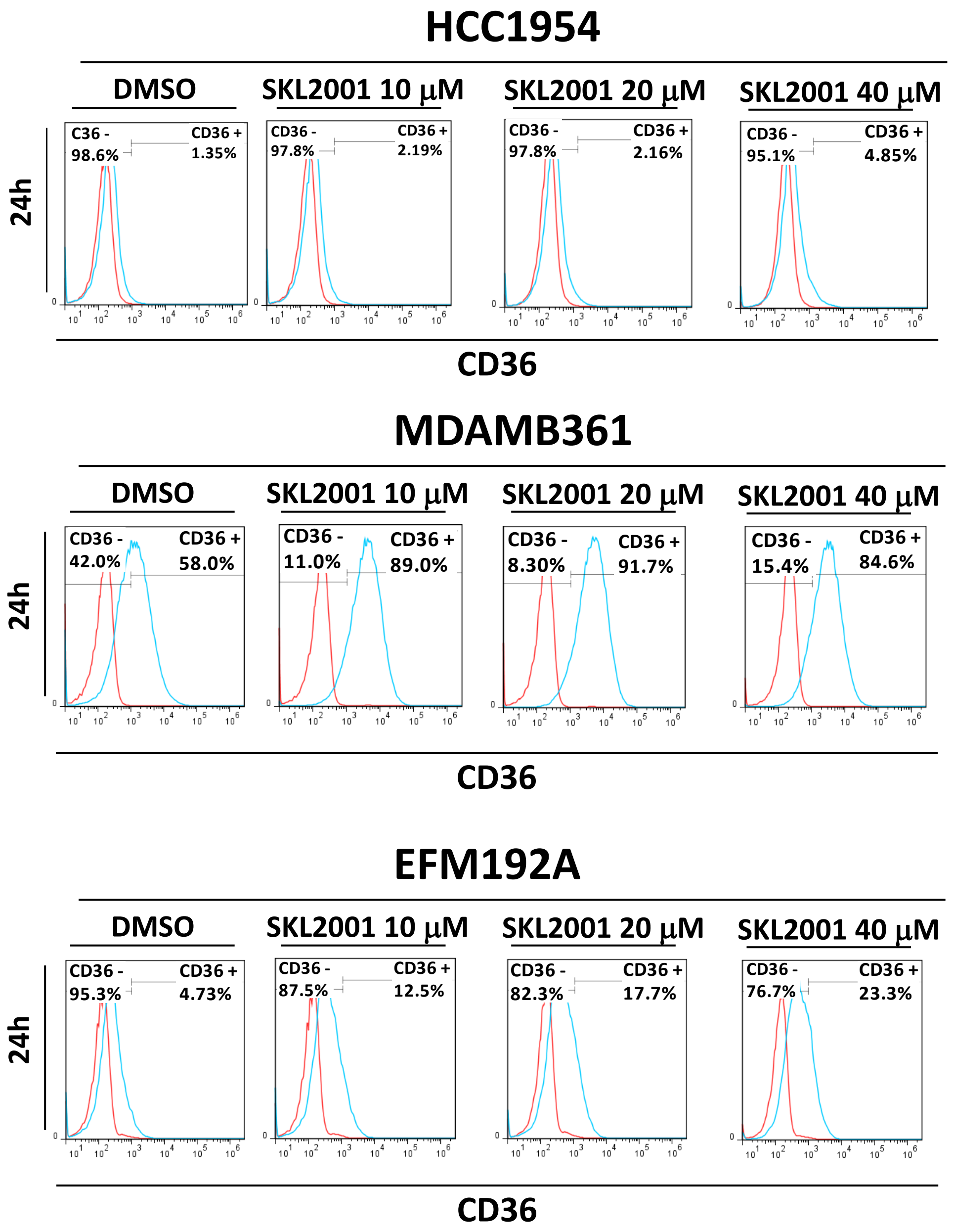

Supplement: Supplementary file 1 — Supplementary Material 1 [file 13046_2025_3276_MOESM1_ESM.pdf]
